# Beyond the Rhizome: Phytochemistry, Biological Activities, and Sustainable Utilization of *Curcuma longa* L.

**DOI:** 10.3390/ph19091480

**Published:** 2026-09-17

**Authors:** Kannika Thongkhao, Siripat Chaichit, Santhosh Kumar Jayanthinagar Urumarudappa, Aekkhaluck Intharuksa

**Affiliations:** 1 School of Allied Health Sciences, Walailak University, Nakhon Si Thammarat 80160, Thailand; kannika.to@wu.ac.th; 2 Functional Materials and Nanotechnology Center of Excellence, Walailak University, Nakhon Si Thammarat 80160, Thailand; 3 Department of Pharmaceutical Sciences, Faculty of Pharmacy, Chiang Mai University, Chiang Mai 50200, Thailand; siripat.chaichit@cmu.ac.th; 4 Department of Crop Physiology, School of Ecology and Conservation, University of Agricultural Sciences, Gandhi Krishi Vigyana Kendra, Bangalore 560065, India; santhu.ju@gmail.com; 5 Department of Post Graduate Studies and Research in Biotechnology, Jnanasahyadri, Kuvempu University, Shimoga 577451, India

**Keywords:** bioavailability, *Curcuma*, curcumin, essential oils, metabolomics, nutraceuticals, turmeric, whole-plant biorefinery, Zingiberaceae

## Abstract

*Curcuma longa* L. (turmeric) is an economically and medicinally important species widely utilized as a spice, traditional medicine, and source of bioactive compounds. Although research and commercial exploitation have predominantly focused on the rhizome and its major curcuminoids, evidence increasingly shows that other plant parts also contain chemically diverse and biologically active constituents. This review integrates current knowledge of the botany, traditional uses, phytochemistry, pharmacological activities, industrial applications, and sustainable utilization of *C. longa*, with particular emphasis on whole plant utilization. Unlike previous reviews that have largely focused on the rhizome and curcuminoids, this review synthesizes evidence across underutilized plant organs and links their tissue-specific phytochemistry with biological activities, industrial applications, and circular-bioeconomy potential. Rhizomes are rich in curcuminoids and turmerone-dominated essential oils, whereas leaf, flower, root, and root tuber contain diverse phenolics, flavonoids, terpenoids, and other specialized metabolites. However, these non-rhizome tissues remain substantially less studied, and their distinct phytochemical and functional value has not yet been systematically established. Preclinical studies have linked these constituents to antioxidant, anti-inflammatory, antimicrobial, antiviral, anticancer, antidiabetic, hepatoprotective, and wound-healing effects. However, clinical evidence remains variable across these activities. Beyond therapeutic applications, underutilized aerial parts and processing residues may serve as source of essential oils, natural preservatives, fibers, biodegradable materials, fermentation products, and bioenergy. Cascading biorefinery approaches that integrate tissue-specific phytochemistry may broaden turmeric utilization beyond the rhizome, reduce agricultural waste, and contribute to circular-bioeconomy development. Further research should prioritize underexplored plant organs, standardized phytochemical characterization, safety assessment, well-designed clinical studies, and techno-economic and life-cycle assessments.

## 1. Introduction

Turmeric (*Curcuma longa* L.) is a perennial herb belonging to the genus *Curcuma* (Zingiberaceae). Native to South Asia, particularly India, it has been cultivated and utilized for centuries in food, medicine, and ritual practices [1,2]. The plant is one of the most extensively studied and commercially exploited species within the genus, owing to its profound ethnopharmacological relevance, culinary importance, and broad industrial applications in nutraceuticals, pharmaceuticals, cosmetics, and food industries [3,4]. Traditional medical systems such as Ayurveda, Siddha, Unani, Thai and Traditional Chinese Medicine (TCM) have long recognized the therapeutic potential of *Curcuma* species. The medicinal use of turmeric was first documented in the Atharveda and later in classical texts such as the Compendium of Materia Medica (Bencao Gangmu) in China [5]. Rhizomes are prescribed for wound healing, digestive disorders, inflammatory conditions, respiratory ailments, liver diseases, dermatological problems, and metabolic complications. In Ayurveda, turmeric is classified as a “Rasayana” herb, denoting rejuvenating and immunomodulatory properties [6]. Beyond medicinal use, turmeric serves as a natural spice, coloring agent, preservative, and cosmetic ingredient, reflecting its deep integration into cultural traditions across South and Southeast Asia [4].

Modern pharmacological studies have validated many of these traditional claims, identifying curcuminoids (curcumin, demethoxycurcumin, bisdemethoxycurcumin) and volatile oils (ar-turmerone, α-turmerone, β-turmerone, curlone, zingiberene) as the principal bioactive constituents. These compounds exhibit diverse pharmacological activities, including antioxidant, anti-inflammatory, antimicrobial, anticancer, hepatoprotective, neuroprotective, and metabolic regulatory effects [7]. Curcumin, the most abundant curcuminoid, modulates multiple signaling pathways such as NF-κB, Nrf2, MAPK, and PI3K/Akt, underpinning its pleiotropic pharmacological actions [8]. While rhizomes have been the primary focus of scientific investigation, *Curcuma* flower remains comparatively underexplored. Emerging evidence indicates that floral tissues contain distinct phytochemical profiles rich in phenolics, flavonoids, anthocyanins, carotenoids, and volatile terpenoids [9]. In several Asian countries, particularly Thailand and Northeast India, young inflorescences and floral bracts are consumed as vegetables or incorporated into traditional foods, underscoring their nutritional and cultural significance [3]. Flower also possesses ornamental value and are increasingly explored for applications in cosmetics and nutraceutical industries. Advances in metabolomics have revealed tissue-specific chemical compositions between flower and rhizome, suggesting differential pharmacological activities and offering new opportunities for sustainable utilization of floral biomass [10].

Beyond turmeric’s traditional use as a spice and medical plant, it functions as an important renewable agricultural crop that serves as a valuable feedstock for bio-based industries. Turmeric provides raw materials for producing natural chemicals in textile industry, food ingredients, pharmaceuticals, cosmetics, biomaterials, including bioenergy. Almost every part of the plant, rhizome, leaf and processing residues, can be utilized and support a circular bioeconomy with minimal waste. Turmeric is increasingly recognized as a promising biorefinery feedstock due to its rich composition of high-value phytochemicals and biomass. Turmeric is well suited as an emerging biorefinery feedstock because nearly every component of the plant can be valorized into high-value products, thereby minimizing waste and aligning with the principles of a circular bioeconomy. Countries in Southeast Asia are important producers of turmeric, where the crop is widely cultivated for food, traditional medicine, and industrial applications. India being the global leader in terms of cultivation area and production, while the plant is distributed across India and Southeast Asian countries, including Bangladesh, Cambodia, Thailand, China, Malaysia, Indonesia, and the Philippines [11]. Despite extensive research on *C. longa*, the available literature remains predominantly rhizome-centered, while evidence on leaf, flower, root, and processing by-products is comparatively fragmented. Importantly, a comprehensive synthesis integrating whole-plant phytochemistry, pharmacological activities, and sustainable utilization, particularly within a biorefinery and circular-bioeconomy framework, remains lacking. This gap limits an integrated understanding of how underutilized plant parts and processing residues could complement conventional rhizome-based utilization.

This review aims to critically summarize and integrate the current knowledge on *C. longa*, with emphasis on its botanical characteristics, traditional and ethnomedicinal uses, phytochemical composition, pharmacological activities, molecular mechanisms, and industrial applications. In particular, it integrates evidence across rhizome, leaf, flower, root, root tuber, and processing by-products to provide a whole-plant perspective linking phytochemical diversity with biological activities and potential value-added utilization. In addition, this review outlines future perspectives for whole-plant valorization, biorefinery integration, and bioeconomy-driven sustainable utilization of *C. longa*.

## 2. Results

### 2.1. Botanical Overview of C. longa

#### 2.1.1. Taxonomy

*C. longa* (turmeric), a triploid species (2n = 3x = 63), is a perennial rhizomatous herb belonging to the family Zingiberaceae (ginger family) and the order Zingiberales. The species is currently recognized under the accepted scientific name *Curcuma longa* L. The genus *Curcuma* (Zingiberaceae) comprises more than 130 species distributed across tropical and subtropical Asia, representing one of the most economically, culturally, and medicinally significant groups of aromatic plants. Species within this genus are cultivated widely in India, Southeast Asia, China, and Indonesia, where they have been utilized for centuries in food, medicine, and ritual practices [1,2].

#### 2.1.2. Morphological Characteristics (Table A1)

*C. longa* is a perennial herbaceous plant with a height of approximately 1.0–1.5 m. It is classified within the genus *Curcuma*, which includes several species such as *C. zanthorrhiza*, *C. zedoaria*, and others that share morphological similarities. Morphological variation across cultivars reflects both genetic diversity and environmental adaptation, and these traits are of considerable importance for taxonomy, ethnobotany, and commercial utilization [12]. The plant typically produces underground rhizomes that serve as the primary medicinal and commercial organs, while aerial part include large distichous leaf and terminal inflorescences borne on separate peduncles. Floral bracts exhibit diverse pigmentation ranging from green to white, pink, and purple, contributing to ornamental value and taxonomic differentiation [13]. Such morphological diversity not only aids species delimitation within the genus *Curcuma* but also underpins its ethnobotanical uses and industrial applications, linking structural traits with phytochemical composition and biomass potential.

**Figure 1:**
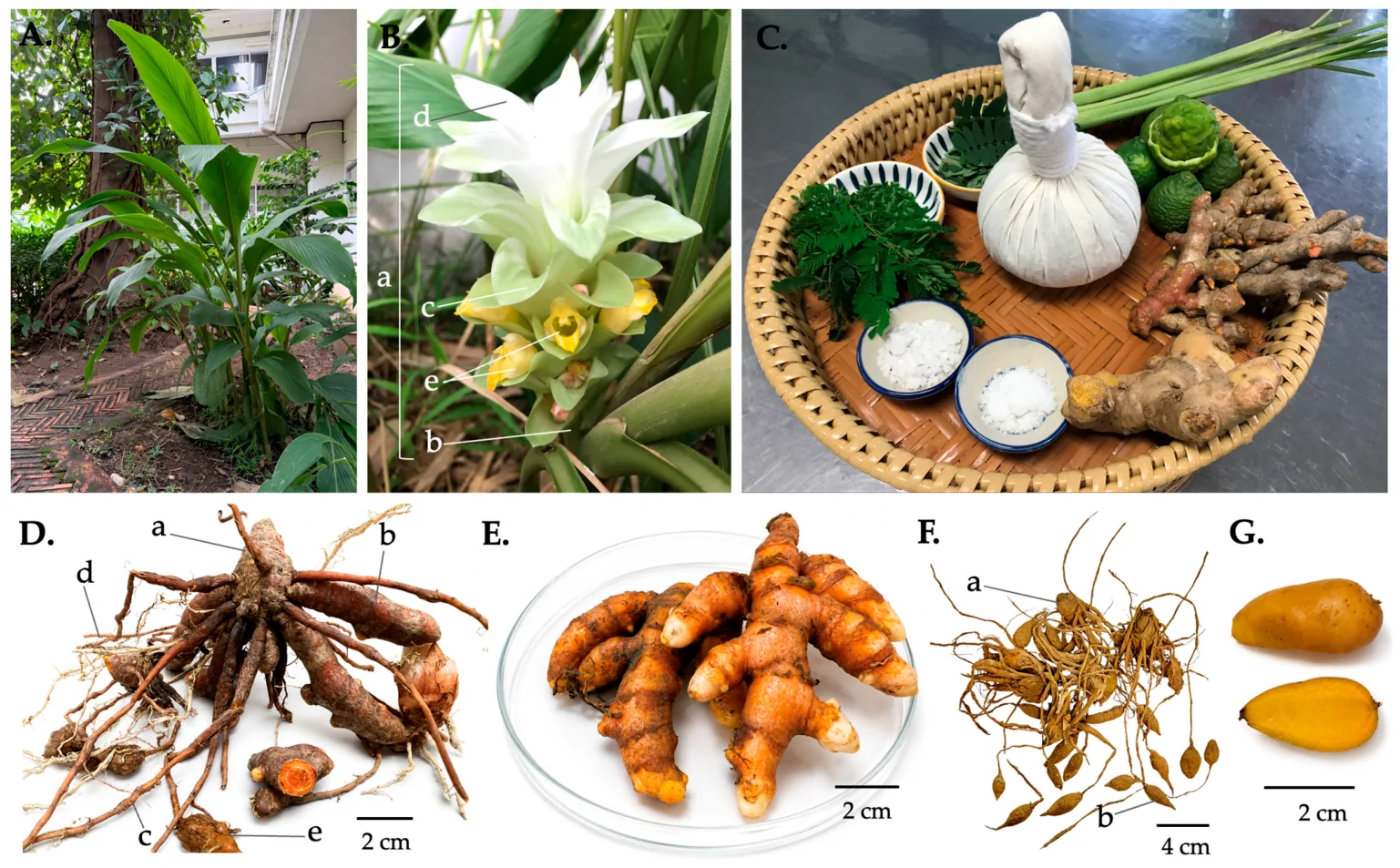
Morphological characteristics and representative traditional uses of *C. longa* L. (**A**) Whole plant of *C. longa* showing the characteristic leafy pseudostem; (**B**) inflorescence with pale green to white bracts and yellow flowers (a. inflorescence spike, b. peduncle, c. fertile bract, d. coma bract, e. flowers); (**C**) representative traditional herbal preparation (Thai massage ball) containing turmeric rhizome together with other herbal ingredients; (**D**) underground rhizome system showing the primary and lateral rhizome (a. main rhizome, b. lateral rhizome, c. ordinary fibrous root, d. adventitious fibrous root, e. root tuber) with a scale bar 2 cm; (**E**) freshly harvested turmeric rhizome, with a scale bar 2 cm; (**F**) fibrous root system with terminal root tubers (a. rhizome, b. root tuber), with a scale bar 4 cm; and (**G**) external and longitudinally sectioned root tuber, with a scale bar 2 cm.

1. Morphology of Underground Parts
  - Rhizome
    The underground rhizome system of *C. longa* comprises two distinct types: the primary “mother” rhizome and secondary lateral branches, commonly referred to as “fingers” (Figure 1E,F). The mother rhizome is typically ovate to ellipsoid (2.5–7.0 cm long, 1.5–4.0 cm wide), whereas lateral branches are cylindrical to slightly compressed (2.5–12.0 cm long, 1.0–2.5 cm diameter), with dimensions varying according to cultivar and environmental conditions [14,15]. Rhizomes are distinctly aromatic due to volatile sesquiterpenoids, particularly turmerones, and exhibit bright yellow to deep orange pigmentation derived from curcuminoids (curcumin, demethoxycurcumin, bisdemethoxycurcumin) and carotenoids. This pigmentation is widely recognized as a key quality marker for commercial turmeric and is closely linked to phytochemical composition. The mother rhizome produces lateral fingers from axillary buds, which constitute the principal medicinal and commercial parts harvested for spice, nutraceutical, and pharmaceutical applications. Post-harvest, rhizomes undergo boiling, drying, and polishing to yield turmeric powder, a process that influences curcuminoid stability and essential oil retention. Recent reviews of Curcumae Longae Rhizoma have highlighted the relationships among botanical source, processing, chemical composition, and pharmacological activity, emphasizing the importance of these factors for quality evaluation and reproducibility [16,17].
  - Root and Root tuber
    The underground system of *C. longa* consists of a highly branched rhizome associated with numerous adventitious, fibrous roots arising from the basal regions of the rhizome (Figure 1D,F) [15,18,19]. The ordinary roots are slender to filiform and extend into the surrounding soil, where they primarily contribute to water and nutrient uptake. These roots can be clearly distinguished from the thickened rhizome, which is an underground stem characterized by nodes, internodes, scale-leaf scars, buds, and lateral branches [18,20]. Some adventitious roots may become distinctly swollen toward their distal ends, forming fleshy storage structures commonly described as tuberous root or root tuber (Figure 1F,G) [15,19]. In the genus *Curcuma*, the roots are generally fibrous and frequently terminate in root tuber, which may be ovoid, ellipsoid, fusiform, or otherwise locally enlarged, depending on the species [19]. These swollen structures remain connected to the rhizome by a comparatively narrow fibrous portion. Unlike rhizomes, root tuber lack node, internode, scale leaf, and axillary bud, confirming their root rather than stem origin [20]. They principally serve as storage organs for water and reserve carbohydrates, thereby supporting plant survival during seasonal dormancy and subsequent vegetative growth [20]. In botanical terminology, the term “nodulose” refers to roots bearing localized knot-like or nodular swellings. However, for *C. longa*, the expressions “roots tuberous at the tip” or “terminally swollen tuberous roots” are more precise than “nodulose roots.” World Flora Online describes *C. longa* as having highly branched, cylindrical, orange or bright-yellow aromatic rhizomes, with roots becoming tuberous at their tips [15]. Regional floras, however, are not fully consistent in their descriptions of tuberous-root formation in *C. longa*. Some sources recognize swollen or root tuber, whereas others describe only the rhizome and adventitious roots; therefore, this character should be interpreted cautiously rather than treated as universally diagnostic of the species. Nevertheless, the occurrence and degree of root-tuber development may vary among specimens or cultivated accessions. Notably, the taxonomic treatment in the Flora of Thailand describes the branched rhizome of *C. longa* in detail but reports that root tuber was not observed in the examined specimens [13]. These differences may reflect variation among specimens or cultivated accessions, as well as differences in taxonomic treatment or morphological terminology among regional floras.
2. Morphology of Arial Parts
  - Leaf
    The leaf of *C. longa* arise basally from the rhizome and are arranged spirally around a pseudostem, which is formed by the tightly overlapping leaf sheaths, a characteristic feature of the family Zingiberaceae (Figure 1A). The leaf sheath encloses adjacent sheaths to form the pseudostem, providing structural support for the aerial portion of the plant. Depending on the genotype and environmental growing conditions, each plant typically produces between 8 and 15 leaves during its vegetative growth. Variation in leaf size, shape, and coloration is influenced by cultivar and environment; some cultivars exhibit purplish midribs or petiole bases, traits used in chemotaxonomic differentiation. Each leaf is composed of three distinct parts: the leaf sheath, petiole, and leaf blade (lamina). Although substantial variation in leaf size has been reported among cultivars and under different environmental conditions, they are oblong to lanceolate, typically 30–90 cm long and 10–25 cm wide, with elongated petioles often exceeding the lamina length [13]. The lamina is dark green adaxially and lighter abaxially, with a smooth texture and prominent midrib. It possesses an entire margin, an acuminate apex, and an attenuate base, while both the adaxial and abaxial surfaces are glabrous. *C. longa* exhibits parallel venation, with numerous longitudinal veins extending continuously from the leaf base to the apex. These veins are interconnected by fine transverse veinlets, which enhance both mechanical strength and the efficiency of water and nutrient transport throughout the lamina. The pattern of parallel venation represents an important diagnostic character in the taxonomy of the family Zingiberaceae. Leaf coloration also shows considerable variation among cultivars. The adaxial (upper) surface is typically bright to dark green, whereas the abaxial (lower) surface is lighter green. In some cultivars, distinctive reddish-purple pigmentation occurs along the midrib, accompanied by a purple tinge near the leaf base or a darker green lamina. These pigmentation patterns are widely recognized as useful morphological descriptors for germplasm characterization and cultivar identification.
  - Inflorescence and Flower
    The inflorescence of *C. longa* (Figure 1B) arises as a terminal spike on a separate, leafless peduncle (10–30 cm, occasionally up to 50 cm). The spike is cylindrical to conical (8–15 cm long, 3–6 cm wide) and consists of overlapping bracts arranged spirally along the rachis. The lower bracts are green and fertile, whereas the upper coma bracts are conspicuous and sterile, ranging from pale green to white, pink, purple, or violet, and function as pollinator attractants [21]. The flower is zygomorphic and bisexual, typically pale yellow, white, pink, or purple, and comprise a tubular calyx, a three-lobed corolla, a prominent labellum (lip), lateral staminodes, a single fertile stamen, and a gynoecium with an inferior ovary and capitate stigma. Flowering occurs seasonally, often during the monsoon or early autumn, depending on geographic condition [13,21].

### 2.2. Traditional Uses

The genus *Curcuma*, particularly *C. longa* L., has been deeply embedded in cultural, medicinal, and spiritual practices across Asia for millennia. Traditional medical systems have systematically applied different parts of the plant, most notably the rhizome and, to a lesser extent, the flower, for a wide spectrum of therapeutic purposes. These ethnomedicinal applications provide the foundation for modern pharmacological investigations and highlight the continuity between traditional knowledge and contemporary science. In Ayurveda, turmeric is classified as a Rasayana herb, denoting rejuvenating and immunomodulatory properties. Classical texts are attributed to turmeric actions such as krimighna (antimicrobial), vranaropana (wound healing), tvagdoshahara (skin disorder remedy), and raktashodhaka (blood purifier) [22]. Topical pastes prepared from fresh or powdered rhizome are applied to wounds, burns, and infections, while decoctions, powders, and medicated ghee are administered internally for digestive disorders, respiratory complaints, febrile conditions, and arthritis [23]. In TCM, turmeric (Jiang Huang) is classified as a bitter, pungent, and warm herb that acts on the liver and spleen meridians. It is primarily indicated for invigorating blood circulation, relieving pain due to blood stasis, and treating hepatobiliary disorders [24]. In Unani medicine, turmeric is considered a diuretic (Mudirr-e-Bawl), liver tonic (Muqawwi-e-Jigar), and antiseptic (Daf-e-Taffun), prescribed for hepatitis, urinary tract infections, and inflammatory conditions [25]. Across Southeast Asia, turmeric rhizomes are employed as antimicrobial agents, blood purifiers, and postpartum tonics. In Malaysia and Indonesia, decoctions are traditionally given to women after childbirth to promote uterine involution and prevent puerperal infections, while in Thailand turmeric is incorporated into remedies for dyspepsia and peptic ulcers [21].

In Thai traditional medicine, turmeric (Khamin Chan) holds a central role beyond digestive and inflammatory disorders. Fresh rhizome paste is applied to insect bites, boils, and minor wounds, while decoctions are used for gallstones, kidney stones, and postpartum recovery. Turmeric is also prescribed for skin conditions, rheumatism, and as a general tonic to restore vitality. Importantly, modern Thai herbal policy has designated turmeric as a flagship herb, promoting its use in medicines, cosmetics, and dietary supplements (Figure 1C), and positioning it as a key economic crop within Thailand’s national bioeconomy strategy [26]. Beyond its therapeutic applications, turmeric has long been integrated into daily life and cultural practices. It functions as a natural preservative by inhibiting lipid peroxidation and microbial growth, as a spice essential to South Asian cuisine, and as a dye for textiles and ritual threads. In Hindu ceremonies, turmeric paste is applied as a symbol of purification, fertility, and prosperity [14].

### 2.3. Phytochemistry of C. longa

*C. longa* is valued as both a culinary spice and a medicinal plant. Its bioactive constituents show marked tissue specific variation between rhizome, the primary medicinal organ and the comparatively underexplored flower [27]. Rhizome contain curcuminoids (2–5% dry weight), while the major compounds are curcumin (70–85%), demethoxycurcumin (10–15%), and bisdemethoxycurcumin (3–5%) [28]. Curcumin and curcuminoid-rich turmeric extracts have demonstrated antioxidant and anti-inflammatory activities and may promote wound repair in experimental studies [29,30]. Advanced metabolomic analyses (UHPLC-Q-Orbitrap HRMS, DESI MSI) reveal curcuminoid localization within epidermal and vascular tissues [31]. Essential oils are dominated by sesquiterpenoids and monoterpenoids, notably ar-turmerone (7.3–38.6%), α/β-turmerone, curlone (1.5–15.9%), zingiberene, and β-curcumene (up to 24.5%), contributing to aroma and pharmacological activity [32]. Additional volatile compounds include α-phellandrene, sabinene, 1,8-cineole, borneol, limonene, and linalool. Flavonoids (quercetin, apigenin), phytosterols (β-sitosterol, stigmasterol), polysaccharides (ukonans), and phenolic acid (caffeic acid) which supporting immunomodulatory and antioxidant functions are also reported in rhizome [33]. Floral essential oils display a distinct volatile profile dominated by 26% p-cymene, 7.6% terpinolene, and 4.1% 1,8-cineole, with minor monoterpenes including linalool, camphor, and borneol [34,35]. Nano emulsion formulations of these volatiles exhibit notable repellent activity, underscoring their potential in vector control applications [36]. Leaf contains diverse range of phytochemical constituents such as flavonoid glycosides and volatile terpenoids. Ethanolic extract from aerial parts of *C. longa* produce numerous flavonol glycosides and dihydroflavonol glucosides [37]. Leaf essential oil is characterized by a complex mixture of monoterpenes and other volatile constituents including 1,8-cineole, α-phellandrene, pinenes, limonene, and cymene derivatives [38]. The chemical profile of the leaf essential oil varies among geographical origins and plant varieties, with different chemotypes showing distinct predominant constituents [38,39]. This phytochemical diversity highlights the potential of *C. longa* leaf as an underutilized source of bioactive compounds beyond the traditionally exploited rhizome. Flower and root tuber remain among the least characterized organs of *C. longa*. Available studies indicate that floral tissues contain phenolics, flavonoids, anthocyanins, carotenoids, and volatile terpenoids, whereas root tuber contains selected mono- and sesquiterpenoids together with other less extensively characterized constituents. However, current evidence is insufficient to define consistent organ-specific chemical markers or to determine whether these tissues provide distinct phytochemical advantages over the rhizome. Comparative metabolomic studies across developmental stages, cultivars, and harvest periods are therefore needed, particularly for flowers whose biomass availability may be seasonally constrained. A comprehensive list of identified phytochemicals is provided in Table 1.

Overall, current phytochemical knowledge of *C. longa* remains strongly centered on the rhizome, particularly curcuminoids and turmerone-rich volatile oils. In contrast, leaf, flower, root, and root tuber have been investigated less systematically, and available reports suggest organ-specific chemical profiles that remain incompletely characterized. This imbalance highlights the need for comparative, organ-specific metabolomic studies to determine whether non-rhizome tissues provide distinct chemical value beyond that of the rhizome.

### 2.4. Pharmacological Activities

**Figure 2:**
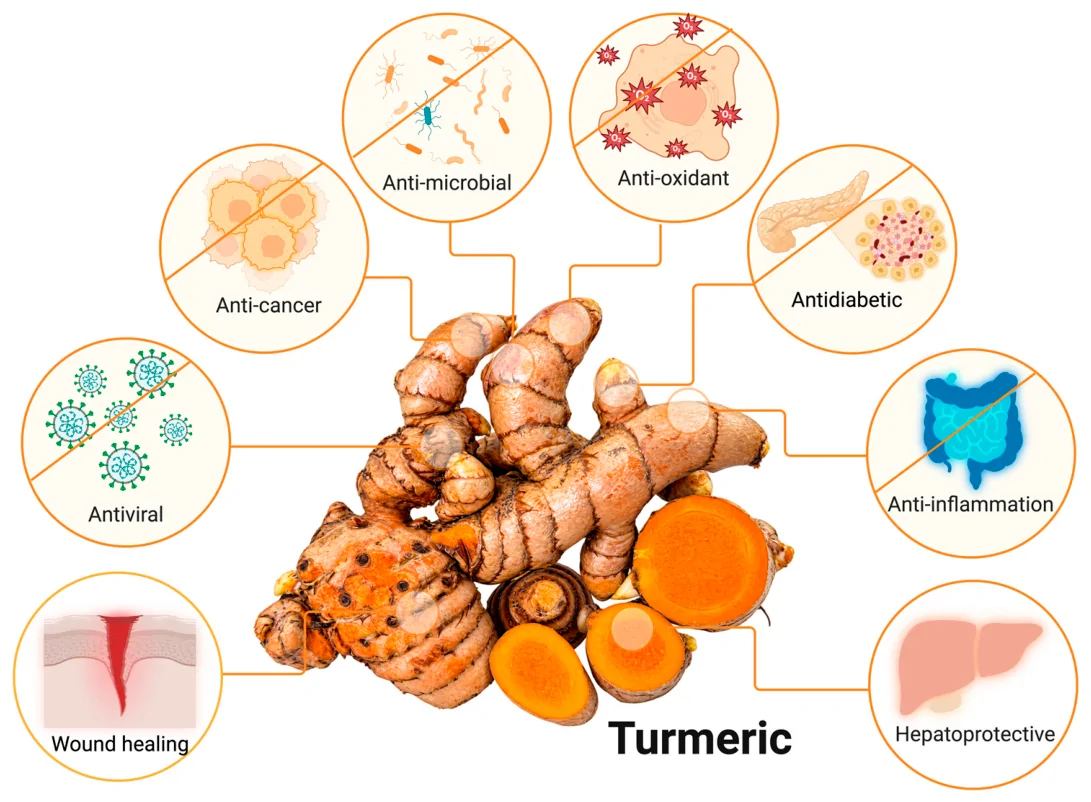
Pharmacological activities of *C. longa*. Created in BioRender. Aekkhaluck Intharuksa. (2026) https://BioRender.com/igdbmrq (accessed on 25 August 2026).

The pharmacological profile of *C. longa* rhizome encompasses a broad spectrum of bioactivities mediated by its diverse phytochemical constituents. These include antioxidant, anti-inflammatory, antimicrobial, anticancer, hepatoprotective, antidiabetic, wound-healing, neuroprotective, and immunomodulatory effects. All pharmacological activities are summarized in Figure 2, which provides an integrated visual overview of its major therapeutic mechanisms and biological targets. Beyond the rhizome, other plant parts, including leaf, flower, and processing residues, contain bioactive phytochemicals with diverse pharmacological properties. Although curcumin and curcuminoids are the best-characterized modulators of NF-κB, Nrf2, MAPK, and PI3K/Akt signaling, mechanistic evidence for turmerones, flavonoids, and other non-curcuminoid constituents remains more limited and heterogeneous; therefore, their pathway-specific similarities and differences cannot yet be defined with confidence. These pharmacological effects should not be viewed as independent phenomena, because oxidative stress, inflammation, immune dysregulation, metabolic disturbance, infection, and abnormal cell proliferation are closely interconnected. The following subsections therefore highlight both the individual activities of *C. longa* and the mechanistic links among them.

#### 2.4.1. Antioxidant Activity

Flower, leaf, and rhizome of *C. longa* exhibit pronounced antioxidant properties, largely attributable to their phenolic compounds, flavonoids, curcuminoids, and volatile terpenoids. Curcumin, the principal curcuminoid, functions as a potent free-radical scavenger and modulator of oxidative-stress-related pathways [78]. Mechanistically, curcumin can neutralize reactive oxygen and nitrogen species and suppress oxidative chain reactions, including lipid peroxidation, while simultaneously strengthening endogenous antioxidant defenses through modulation of glutathione and antioxidant enzymes such as glutathione peroxidase, superoxide dismutase (SOD), and catalase [79,80]. Curcumin also activates the nuclear factor erythroid 2-related factor 2 (Nrf2) pathway, facilitating Nrf2 nuclear translocation and the subsequent transcriptional upregulation of antioxidant proteins and phase II detoxification enzymes [81]. The rhizome contains curcumin, demethoxycurcumin, and bisdemethoxycurcumin as its principal curcuminoids, typically accounting for approximately 77–80%, 12–17%, and 3–6% of the curcuminoid fraction, respectively [1,82,83]. Comparative studies of the individual compounds have generally shown that antioxidant capacity follows the order curcumin > demethoxycurcumin > bisdemethoxycurcumin in phosphomolybdenum and linoleic acid-peroxidation models, although their relative activity may vary according to the assay system [84]. Isolated curcuminoids have also exhibited ABTS radical-scavenging and ferric-reducing activities, and their antioxidant behavior may be modified by complexation with cyclodextrins or phospholipids [83].

In addition to curcuminoids, the antioxidant activity of *C. longa* is supported by phenolic acids, flavonoids, and volatile constituents, particularly turmerones and other mono- and sesquiterpenes. Ethanolic rhizome extracts generally demonstrate stronger antioxidant activity than aqueous extracts, reflecting the greater extraction efficiency of ethanol for phenolic and flavonoid constituents [85]. For example, ethanolic extracts of different turmeric varieties exhibited marked DPPH radical-scavenging and ferric-reducing antioxidant power (FRAP), although the magnitude of activity varied according to variety, geographical origin, and extract composition [85]. Rhizome essential oil containing ar-turmerone, α-turmerone, and β-turmerone also showed measurable DPPH, FRAP, and CUPRAC activities, with a CUPRAC value of 3.40 ± 0.071 mmol Trolox equivalents/g oil and 51.45 ± 0.59% DPPH scavenging at 150 µg/mL [86]. Another turmeric essential oil dominated by α-turmerone, β-turmerone, and ar-turmerone exhibited ABTS and DPPH scavenging activities with IC_50_ values of 0.54 and 10.03 mg/mL, respectively [87]. Beyond the rhizome, *C. longa* leaf represents an underutilized source of antioxidant compounds. Leaf extracts have been reported to inhibit oxidative processes, including lipid oxidation, while their antioxidant activity varies according to the extraction solvent [50,88]. The leaf essential oil, characterized predominantly by α-phellandrene, 2-carene, and eucalyptol, showed strong scavenging effects against DPPH, ABTS, and hydrogen peroxide radicals, with IC_50_ values of 8.62 ± 0.18, 9.21 ± 0.29, and 4.35 ± 0.16 µg/mL, respectively [39]. The flower of *C. longa* has likewise demonstrated antioxidant potential, with flower extracts showing pronounced DPPH radical-scavenging activity and considerable phenolic contents [89]. Overall, these findings indicate that antioxidant activity in *C. longa* is not attributable to curcumin alone but arises from the combined actions of curcuminoids, phenolics, flavonoids, and volatile terpenoids, whose relative contributions depend on the plant part, cultivar, processing conditions, extraction solvent, and analytical model employed [80,85,86]. However, most of the available evidence for the antioxidant activity of *C. longa* is derived from in vitro chemical or cell-free assays, whereas direct in vivo and clinical evidence specifically linking these antioxidant effects to meaningful health outcomes remains comparatively limited. Interpretation should therefore be cautious because differences in assay conditions, extract composition, and test concentrations, together with the low systemic bioavailability of curcumin, may limit the direct translation of these findings to physiological or clinical settings. Direct comparison among antioxidant studies should be interpreted cautiously because DPPH, ABTS, FRAP, CUPRAC, ORAC, and related assays measure different aspects of antioxidant behavior and are influenced by assay conditions, solvent systems, reaction time, reference standards, and reporting units. Consequently, differences in IC_50_ values or antioxidant capacity across studies may reflect methodological variation as well as true differences in extract composition or biological activity. Results obtained using different analytical models should therefore be considered complementary rather than directly interchangeable, and stronger conclusions require standardized experimental conditions and, where possible, the use of multiple antioxidant assays. The antioxidant and anti-inflammatory effects of *C. longa* are mechanistically interconnected, as excessive reactive oxygen species can activate redox-sensitive inflammatory pathways such as NF-κB, while inflammatory signaling can further amplify oxidative stress. The reported antioxidant capabilities of *C. longa* extracts, essential oils, and isolated curcuminoids are summarized in Table 2.

#### 2.4.2. Anti-Inflammatory Activity

*C. longa* and its bioactive constituents exert anti-inflammatory effects through the coordinated modulation of inflammatory, immune, and redox-sensitive pathways. The major constituents include the curcuminoids curcumin, demethoxycurcumin, and bisdemethoxycurcumin, together with non-curcuminoid compounds such as turmeronols A and B, bisacurone, tetrahydrocurcumin, ar-turmerone, α-turmerone, β-turmerone, curdione, polysaccharides, and other volatile terpenoids [92,93,94,95,96]. At the molecular level, curcumin suppresses nuclear factor kappa B (NF-κB) activity by inhibiting IκB kinase phosphorylation, preventing IκB degradation, and reducing the nuclear translocation and DNA-binding activity of the NF-κB p65 subunit. This leads to reduced expression of tumor necrosis factor-α (TNF-α), interleukin (IL)-1β, IL-2, IL-6, IL-8, IL-12, IL-17, interferon-γ, cyclooxygenase-2 (COX-2), inducible nitric oxide synthase, prostaglandin E_2_, nitric oxide, chemokines, and adhesion molecules [92,94,96,97]. Curcumin also interferes with Toll-like receptor and NOD2 signaling, JAK/STAT, AP-1, MAPK, PI3K/Akt/mTOR, and IL-1 receptor-associated kinase pathways while modulating PPARγ, SIRT1, and Nrf2/heme oxygenase-1 signaling [92,97,98,99,100]. Its activity extends beyond direct suppression of soluble mediators to regulation of immune-cell function. Curcumin can inhibit dendritic-cell maturation and decrease CD80, CD86, CD40, MHC class II, ICAM-1, and CD11c expression; attenuate neutrophil recruitment and macrophage production of IL-1β, IL-6, IL-8, IL-12, TNF-α, MCP-1, MIP-1α, and nitric oxide; suppress B-cell activation and immunoglobulin class switching; and modulate Th1, Th2, Th17, regulatory T-cell, CD8^+^ T-cell, and natural-killer-cell responses [97]. These immunomodulatory effects are dose- and context-dependent, as curcumin may suppress pathogenic Th1/Th17 responses and increase IL-10-producing regulatory T cells in autoimmune and allergic models, while lower doses may enhance selected antitumor immune responses [97].

The anti-inflammatory and immunomodulatory mechanisms have been reproduced in numerous in vivo models. In acute lung injury, curcumin reduced inflammatory-cell infiltration, myeloperoxidase activity, IL-6, TNF-α, CCL7, NLRP3 inflammasome activation, caspase-1, IL-1β maturation, and pyroptosis through a SIRT1-dependent mechanism [99]. In ovalbumin-induced asthma, it reduced eosinophilia, Th2 cytokines, IgE, mucus hypersecretion, and airway hyperresponsiveness through PPARγ-dependent suppression of NF-κB and activation of Nrf2/HO-1 [98,100]. Experimental arthritis studies similarly showed reduced synovitis, granulomatous inflammation, paw edema, neutrophil accumulation, cartilage degradation, and bone erosion, accompanied by suppression of IL-1β, TNF-α, MCP-1, COX-2, PGE_2_, MMPs, β-integrins, and other adhesion molecules [94,97]. Curcumin also protected chondrocytes from IL-1β-induced apoptosis and matrix degradation by preserving collagen type II and β1-integrin expression and inhibiting caspase-3, NF-κB, JNK, MAPK, and PI3K/Akt signaling [97]. Broader immunological models indicate activity in inflammatory bowel disease, autoimmune encephalomyelitis, lupus nephritis, allergic inflammation, periodontal disease, hepatic inflammation, pancreatitis, diabetic nephropathy, and immune-complex glomerulonephritis. In these models, curcumin regulated TLR2/TLR4/TLR9, MyD88, NOD2, STAT3, RORγt, Notch1–GATA3, hKv1.3, complement activation, and the balance among Th1, Th2, Th17, and regulatory T cells [97]. In silico studies further predict that curcumin and other *C. longa* constituents may interact with COX-1, COX-2, PDE-4B, SOD, catalase, and glutathione peroxidase, although these computational results require direct biochemical validation [101]. Collectively, the experimental evidence indicates that *C. longa* acts not simply as a COX inhibitor but as a multitarget regulator of innate and adaptive inflammatory responses.

Clinical studies have reported potentially beneficial effects of curcumin and *C. longa* extracts as adjunctive interventions in selected chronic inflammatory conditions, particularly arthritis and metabolic inflammation; however, findings remain heterogeneous and are not uniformly positive across trials. In knee osteoarthritis, curcuminoids and standardized extracts reduced pain, WOMAC or KOOS scores, rescue-medication use, and selected biomarkers such as IL-4, IL-6, hs-CRP, IL-1β, reactive oxygen species, and malondialdehyde [97,102,103,104,105,106,107]. Several trials reported efficacy comparable with diclofenac or other NSAIDs, with fewer gastrointestinal adverse effects, while a curcuminoid–diclofenac combination improved pain and function more than diclofenac alone in one trial [104,106]. However, an earlier double-blind randomized study in primary knee osteoarthritis found that adding 1000 mg/day curcuminoids to diclofenac 75 mg/day for three months produced only a nonsignificant tendency toward greater improvement in pain and daily function compared with diclofenac alone; VAS and all five KOOS domains showed no statistically significant between-group differences [108]. This finding is important because it illustrates that a plausible complementary mechanism—curcumin-mediated downregulation of NF-κB, cytokines, COX-2 expression, adhesion molecules, and MMPs alongside direct enzymatic COX inhibition by diclofenac—does not necessarily translate into a measurable additive clinical benefit at every dose or study design [108]. In broader populations, hot-water extracts containing bisacurone and turmeronols reduced CRP, TNF-α, IL-6, and soluble VCAM-1 in participants with overweight or prehypertension, while trials and meta-analyses in type 2 diabetes reported reductions in CRP or hs-CRP and improvements in selected metabolic markers [109,110,111,112,113,114]. Exercise-related trials remain heterogeneous: some demonstrated reductions in TNF-α, IL-8, CK, myoglobin, muscle injury, or soreness, whereas others reported no significant effect on CRP, IL-1β, IL-6, TNF-α, or other cytokines [113,114,115,116,117,118]. Therefore, the overall evidence supports anti-inflammatory and immunomodulatory potential, but the magnitude of benefit depends on dose, formulation, bioavailability, disease context, treatment duration, and the biomarker or clinical endpoint selected. Nevertheless, translation of these findings into clinical practice remains constrained by the low and formulation-dependent oral bioavailability of curcumin, substantial heterogeneity in extract composition, dosing regimens, treatment duration, and clinical endpoints, and the relatively small sample sizes of many individual trials. Moreover, the concentrations required to modulate inflammatory pathways in experimental systems may not always be achieved in human tissues, emphasizing the need for larger, well-controlled trials using standardized and pharmacokinetically characterized preparations. This interaction between redox regulation and inflammatory signaling also provides a mechanistic bridge to other activities discussed below, because persistent inflammation can alter host defense, metabolic homeostasis, tissue repair, and pathways involved in tumor initiation and progression.

#### 2.4.3. Antimicrobial and Antibiofilm Activities

*C. longa* and its major constituents exhibit antimicrobial activity against a broad range of Gram-positive and Gram-negative bacteria, fungi, mycobacteria, and biofilm-forming pathogens. Nevertheless, the magnitude of activity varies considerably according to the plant part, extraction solvent, chemical composition, formulation, microbial strain, and susceptibility-testing method. Rhizome extracts prepared using ethanol, methanol, ethyl acetate, petroleum ether, hexane, or other non-polar solvents generally inhibited *Staphylococcus aureus*, including methicillin-resistant *S. aureus* (MRSA), *Bacillus* spp., *Enterococcus faecalis*, *Aeromonas hydrophila*, *Edwardsiella tarda*, and several *Vibrio* species. Non-polar fractions were frequently among the most active preparations, probably because of their enrichment in ar-turmerone, α- and β-turmerone, curlone, and other volatile sesquiterpenes. For example, the n-hexane rhizome fraction inhibited multidrug-resistant MRSA, *Pseudomonas aeruginosa*, and *Acinetobacter baumannii*, with MIC values as low as 2.5 mg/mL, while ar-turmerone was identified as the predominant constituent at 34.63% [119]. Similarly, turmeric volatile oil and solvent extracts inhibited *S. aureus*, *Klebsiella ornithinolytica*, and *Citrobacter gillenii*, with the volatile oil showing an MIC of 6.25 mg/mL against *K. ornithinolytica* [120]. Solvent selection therefore influences both the chemical profile and antimicrobial spectrum. Methanol generally yielded the highest curcuminoid content, whereas n-hexane concentrated turmerone and ar-turmerone were reported for the inhibition of *Aspergillus niger* and *A. flavus* [61]. Antimicrobial activity is also present in underutilized aerial tissues. *C. longa* leaf essential oil, dominated by α-phellandrene, 2-carene, and eucalyptol, inhibited *Staphylococus aureus*, *Escherichia coli*, *P. aeruginosa*, and *Salmonella enterica*, supporting the valorization of turmeric leaf as a source of antimicrobial essential oil rather than their disposal as agricultural waste [39].

Purified curcumin showed strain-specific activity. Although Gram-positive organisms were generally more susceptible, MIC values varied from 5 to 15 µg/mL against selected periodontal bacteria to ≥2000 µg/mL against some multidrug-resistant clinical isolates. In an evaluation of more than 100 strains, *Streptococcus pyogenes* had a median MIC of 31.25 µg/mL, whereas MDR isolates of *Staphylococus aureus*, *Staphylococcus haemolyticus*, *E. coli*, and *Proteus mirabilis* commonly showed MICs of ≥2000 µg/mL [121]. In addition to direct growth inhibition, curcumin and turmeric extracts interfere with bacterial adhesion, quorum sensing, virulence-factor expression, extracellular polymeric substances, and mature biofilms. Curcumin inhibited *Porphyromonas gingivalis* at an MIC of 62.5 µg/mL and reduced the expression of adhesion-associated genes (*fimA*, *hagA*, and *hagB*) and gingipain genes (*rgpA*, *rgpB*, and *kgp*) [122]. Whole turmeric extract was more effective than isolated curcumin against *S. aureus* and *P. aeruginosa* biofilms, suggesting cooperative effects among curcumin, dehydrozingerone, methyl palmitate, ar-turmerone, and other constituents [123]. Nanoformulation further improved activity: nanocurcumin produced larger inhibition zones than bulk curcumin against both *S. aureus* and *E. coli*, while curcumin-loaded nanogels disrupted established *S. aureus* and *P. aeruginosa* biofilms [124,125].

Turmeric constituents may also restore or enhance antibiotic susceptibility. Curcumin reduced the MICs of oxacillin, ampicillin, ciprofloxacin, and norfloxacin against MRSA, and its combination with oxacillin reduced viable bacterial counts below the detectable limit after 24 h [126]. Bisdemethoxycurcumin was particularly potent, with MIC values of 7.8–15.6 µg/mL against six MRSA strains. It reduced gentamicin MICs by as much as 16-fold and suppressed mecA transcription and PBP2a expression, indicating interference with a central β-lactam-resistance mechanism [127]. Preliminary clinical evidence also supports local application. In patients with chronic periodontitis, 1% curcumin gel used with scaling and root planning reduced *P. gingivalis*, *Prevotella intermedia*, *Fusobacterium nucleatum*, and *Capnocytophaga* counts for up to six months [128]. Overall, the evidence supports turmeric-derived preparations as potential antimicrobial, antibiofilm, antibiotic-potentiating, food-preservative, and topical therapeutic agents. However, most evidence remains in vitro, and direct comparison across studies is constrained by differences in extraction procedures, microbial strains, inoculum size, formulations, endpoints, and concentration units. The reported antimicrobial activities of *C. longa* extracts, essential oils, curcuminoids, and related formulations are summarized in Table 3.

#### 2.4.4. Antiviral Activity

Experimental evidence indicates that *C. longa* and its major curcuminoids possess antiviral activity against both DNA and RNA viruses through multiple mechanisms, including direct interference with viral particles and modulation of host-dependent replication pathways. Although evidence against DNA viruses remains relatively limited, several studies have demonstrated notable activity against hepatitis B virus (HBV). Curcumin was shown to interfere with HBV attachment and internalization through interaction with the sodium taurocholate cotransporting polypeptide (NTCP), resulting in dose-dependent reductions in extracellular and intracellular viral DNA and covalently closed circular DNA (cccDNA) levels [141]. At 30 µM, extracellular viral load, intracellular HBV DNA, and cccDNA were reduced by approximately 55%, 67%, and 50%, respectively. Curcumin also exhibited post-entry activity by decreasing histone H3 acetylation and reducing the association of acetylated histones H3 and H4 with the HBV cccDNA minichromosome; treatment with 20 µM for two days decreased HBsAg and cccDNA levels by 57.7% and 75.5%, respectively [142]. In addition, direct pre-incubation with curcumin reduced the infectivity of the enveloped DNA viruses pseudorabies virus and vaccinia virus, most likely through disruption of viral-envelope membrane integrity, although quantitative IC_50_ or EC_50_ values were not reported [143].

The available evidence is considerably broader for RNA viruses, encompassing influenza A virus, dengue virus, Zika virus, chikungunya virus, hepatitis C virus (HCV), coronaviruses, enteroviruses, rhinovirus, respiratory syncytial virus, human parainfluenza virus, and HIV-1. Curcumin and related compounds appear to act at multiple stages of the viral life cycle, particularly during viral attachment, entry, and membrane fusion. Direct interaction with viral particles has been demonstrated for influenza A virus, where curcumin reduced infectivity and inhibited haemagglutinin-mediated receptor binding in H1N1 and H6N1 subtypes [144]. Its ability to alter viral-envelope integrity may contribute to a broader activity against enveloped viruses, including influenza virus, Japanese encephalitis virus, dengue virus, pseudorabies virus, and vaccinia virus, whereas the non-enveloped enterovirus 71 was comparatively unaffected by direct virucidal treatment [143]. Similar interference with viral attachment has been observed for Zika and chikungunya viruses [145]. In HCV, curcumin altered envelope membrane fluidity and impaired viral binding, fusion, and cell-to-cell transmission, resulting in inhibition of entry across all major genotypes [146]. Inhibition of viral internalization and virus-mediated membrane fusion has also been demonstrated for porcine reproductive and respiratory syndrome virus [147], while direct virucidal and entry-associated effects have been reported against SARS-CoV-2 [148,149]. Beyond viral entry, curcuminoids can interfere with viral enzymes, genome replication, protein synthesis, and intracellular replication structures. Thirteen curcuminoids isolated from methanolic *C. longa* rhizome extract inhibited neuraminidases of influenza A H1N1 and H9N2 strains, with IC_50_ values of 6.18–40.17 and 3.77–31.82 µg/mL, respectively. Demethoxycurcumin, bisdemethoxycurcumin, and curcumin also retained activity against wild-type and oseltamivir-resistant H1N1 H274Y neuraminidase, suggesting potential activity against drug-resistant influenza variants [150]. The synthetic analogue curcumin A additionally inhibited HIV-1 reverse transcription and showed greater potency than native curcumin in primary peripheral blood mononuclear cells [151]. Host-dependent mechanisms also contribute substantially to antiviral activity. In HCV infection, curcumin induced heme oxygenase-1 and suppressed PI3K–AKT signaling, resulting in reduced viral RNA and protein expression [152]. In enterovirus 71, inhibition of viral RNA synthesis, protein expression, and progeny production was associated with downregulation of GBF1 and PI4KB, which are required for viral replication-complex formation [153]. A related effect was observed in human parainfluenza virus type 3, where disruption of F-actin organization, suppression of PI4KB, and impaired formation of viral inclusion bodies limited viral RNA synthesis [154]. Against influenza A virus, antiviral activity has additionally been associated with modulation of PI3K–AKT, NF-κB/MAPK, and RIG-I-related signaling. AKT1, RELA, MAPK1, and TP53 have been identified as potential molecular targets, and attenuation of influenza-induced AKT expression has been demonstrated in infected MDCK cells [155]. Monoacetylcurcumin similarly suppressed Akt phosphorylation and showed synergistic antiviral activity when combined with curcumin [156]. Bisabolane-type sesquiterpenoids isolated from *C. longa* rhizome also inhibited influenza replication and decreased virus-induced TNF-α, IL-6, IL-8, and IP-10 production through regulation of NF-κB/MAPK and RIG-I/STAT1/2 signaling [157]. Recently, curcumin and demethoxycurcumin were shown to enhance innate antiviral immunity against enterovirus D68 through the CRYAB–RBM26 axis. This mechanism involved stabilization of RBM26, modulation of CMPK2-dependent nucleotide metabolism, restoration of mitochondrial DNA levels, and activation of the cGAS–STING–TBK1–IRF3 pathway, thereby strengthening type I interferon responses and suppressing viral replication. Demethoxycurcumin exhibited greater physicochemical stability and stronger antiviral activity than curcumin in both cell-based and neonatal mouse models [158]. Overall, antiviral evidence is relatively strong at the mechanistic and preclinical levels but remains limited clinically. Most studies are based on cell or animal models, limiting direct clinical extrapolation. Detailed quantitative findings, tested materials, and corresponding RNA viral strains are summarized in Table 4.

#### 2.4.5. Anticancer and Antimetastatic Activities

Beyond direct antiviral effects, modulation of host inflammatory, oxidative, PI3K/Akt, NF-κB, and related signaling pathways illustrates the broader multitarget actions of turmeric constituents. Many of these same pathways are also implicated in abnormal cell proliferation, survival, angiogenesis, and tumor progression. Cancer remains a major therapeutic challenge because tumor progression involves multiple interconnected processes, including uncontrolled proliferation, resistance to apoptosis, angiogenesis, invasion, and metastasis, which has encouraged continued investigation of structurally diverse natural products and their derivatives as potential anticancer agents [163]. Within this context, *C. longa* and its constituents exhibit anticancer effects through simultaneous modulation of cell proliferation, cell-cycle progression, apoptosis, inflammatory signaling, and replicative immortality. Although curcumin is the most extensively investigated constituent, several studies indicate that the total turmeric extract may be more effective than isolated curcuminoids because of interactions among curcumin, demethoxycurcumin, bisdemethoxycurcumin, turmerones, and other minor constituents. Total extracts showed stronger antiproliferative activity than individual curcuminoids against lung, colon, and glioblastoma cancer cells and enhanced the activity of cisplatin in A549 cells [164]. Turmeric also inhibited NF-κB and STAT3 signaling, downregulated Bcl-2, cFLIP, XIAP, c-IAP1, cyclin D1, c-Myc, and CXCR4, induced the death receptors DR4 and DR5, and sensitized cancer cells to capecitabine and paclitaxel [165]. Mechanistically, curcumin promotes mitochondrial apoptosis through Bax/Bak activation, mitochondrial membrane disruption, cytochrome c and Smac/DIABLO release, and activation of caspase-9 and caspase-3 [166]. It also increased p53 and caspase-3 expression in HeLa cervical cancer cells and inhibited telomerase activity in lung and breast cancer models, suggesting additional effects on tumor-suppressor signaling and cellular immortality [167,168,169].

The anticancer activity of turmeric is also strongly associated with suppression of angiogenesis, epithelial–mesenchymal transition, cancer stem-like properties, migration, and invasion. Curcumin reduced mammosphere formation and the expression of Oct4, Nanog, Sox2, vimentin, fibronectin, and β-catenin while increasing E-cadherin in breast cancer cells, indicating inhibition of both stemness and EMT [170]. Its antimetastatic activity further involves inhibition of α6β4-integrin/Akt/ENPP2 and Rac1/PAK1 signaling, resulting in reduced cytoskeletal rearrangement and decreased MMP-2 and MMP-9 expression in breast and lung cancer cells [171,172,173]. Curcumin additionally suppresses angiogenic signaling by decreasing VEGF, angiopoietin-1/2, VEGFR2/KDR, Akt, and ERK activity, thereby inhibiting endothelial-cell proliferation and migration and promoting endothelial apoptosis [174,175]. In lymphoma-bearing mice, long-term curcumin treatment also reduced ROS production, HIF-1α, c-Myc, LDH-A, PKCα, VEGF, and MMP activity, linking suppression of tumor glycolysis and oxidative stress to reduced angiogenesis [176].

Among the most notable findings, turmeric ethanolic extract showed greater efficacy than an equivalent amount of curcumin alone in colorectal cancer models, partly because turmerones enhanced intracellular curcumin accumulation and contributed additional antiangiogenic and immunomodulatory effects [177]. In an orthotopic colorectal cancer model, the extract suppressed primary tumor growth and liver and lung metastases through regulation of cofilin, FAK/Src, Akt, ERK, STAT3, and EMT-related pathways, while also enhancing T-cell responses and modifying the tumor microenvironment [178]. More clinically relevant evidence was obtained from patient-derived colorectal cancer xenografts, in which oral turmeric extract inhibited tumor growth, metastasis, and recurrence, with reported antitumor and antimetastatic response rates of 60% and 83.3%, respectively; these effects were associated with modulation of Wnt/β-catenin, Src, EGFR, Rho, and EMT-related targets [179]. Extraction conditions also influenced potency, as microwave- and ultrasound-assisted methods increased curcuminoid or phenolic recovery and produced extracts with stronger antiproliferative activity than conventional extraction. Nevertheless, the evidence remains predominantly preclinical, and the activity of one chemically standardized preparation should not be generalized to all turmeric products without further clinical validation. However, despite extensive in vitro and in vivo evidence, direct clinical support for the anticancer and antimetastatic efficacy of *C. longa* or curcumin remains limited, and most human studies have focused on safety, pharmacokinetics, biomarker modulation, or adjunctive use rather than definitive effects on tumor regression, metastasis, or survival. Translation of the preclinical findings is further complicated by the relatively high concentrations frequently required in cell-based studies and the poor and formulation-dependent systemic bioavailability of curcumin. More broadly, nanoparticle-based delivery systems have been investigated as a strategy to protect orally administered bioactive agents from degradation and improve their intestinal delivery and bioavailability [180]; however, curcumin-specific formulations require separate pharmacokinetic and clinical validation. The overlap among oxidative stress, chronic inflammation, PI3K/Akt signaling, and metabolic regulation also links the anticancer effects of turmeric constituents with their reported actions on glucose and lipid homeostasis.

#### 2.4.6. Antidiabetic and Metabolic Effects

Evidence from mechanistic and animal studies indicates that *C. longa* and its constituents regulate glucose homeostasis through multiple complementary pathways. Turmeric aqueous extract stimulated insulin secretion from pancreatic tissue and increased glucose uptake by skeletal muscle through a wortmannin-sensitive pathway, suggesting involvement of PI3K/Akt-mediated insulin signaling [181]. Curcuminoids and sesquiterpenoids, including curcumin, desmethoxycurcumin, bisdesmethoxycurcumin (BDMC), and ar-turmerone, also exhibited PPARγ ligand-binding activity and suppressed hyperglycemia in genetically diabetic KK-A^y^ mice, indicating that both volatile and non-volatile turmeric constituents may act additively or synergistically to improve insulin sensitivity [182]. BDMC provides an additional insulin-independent mechanism by inhibiting pancreatic α-amylase and delaying starch digestion. This activity was demonstrated both in vitro and in diabetic rats, in which oral BDMC reduced the glucose response to starch and improved serum glucose, fructosamine, amylase, and other metabolic parameters [183,184,185]. Recent findings suggest that curcumin may additionally inhibit DPP-4, PTP1B, intestinal α-amylase, and lipase, while increasing glycogen synthase activity and hepatic glycogen storage, thereby influencing incretin signaling, insulin sensitivity, carbohydrate digestion, and lipid absorption simultaneously [186].

Curcumin also preserves pancreatic β-cell structure and function by attenuating oxidative stress, inflammation, endoplasmic-reticulum stress, and apoptosis. In diabetic models, curcumin reduced fasting glucose and HbA1c, improved glucose tolerance, increased insulin secretion, activated Nrf2/HO-1 and PI3K/Akt signaling, restored GLUT2 or GLUT4 expression, and suppressed NF-κB, TNF-α, IL-1β, IL-6, Bax, and caspase-dependent apoptotic pathways [187,188,189]. Turmeric and curcumin further reduced flux through the polyol pathway, lipid peroxidation, and sorbitol dehydrogenase activity in alloxan-induced diabetic rats, suggesting a potential role in limiting oxidative and glycation-related diabetic complications [190]. Histological evidence showed protection of pancreatic islets, reduced inflammatory infiltration, and the appearance of small newly formed islets following prolonged treatment. A water-soluble curcumin derivative also promoted the progressive recovery of insulin- and C-peptide-producing islets in a long-term type 1 diabetic model, although these regenerative findings remain preclinical [47,191]. Nanoformulations generally produced stronger metabolic and pancreatic effects than conventional curcumin. Nanocurcumin increased insulin, glucokinase, glycogen synthase, insulin-receptor, and GLUT2 expression with effects comparable to gliclazide, while curcumin nanoparticles reduced pro-inflammatory cytokines and oxidative stress and restored insulin immunoreactivity in pancreatic β-cells [192,193]. Targeted and nanoparticle-based delivery is therefore considered a promising strategy for overcoming curcumin’s poor absorption and directing its anti-inflammatory activity toward macrophages and insulin-responsive tissues [194].

Clinical studies have reported modest improvements in selected glycemic and metabolic outcomes, although the findings vary substantially according to participant characteristics, formulation, dose, and treatment duration. In healthy participants, a single 6 g dose of turmeric increased postprandial insulin secretion without altering plasma glucose or glycemic index, indicating an insulinotropic effect that may be more apparent before overt metabolic dysfunction develops [195]. In patients with type 2 diabetes, turmeric combined with piperine for 120 days reduced fasting glycemia, HbA1c, HOMA-IR, and triglycerides [196], while a 12-month curcumin intervention improved fasting glucose, HbA1c, β-cell function, insulin resistance, adiponectin, leptin, and body mass index [197]. Turmeric supplementation also reduced glucose, insulin, HOMA-IR, and leptin in patients with nonalcoholic fatty liver disease, whereas curcumin lowered fasting glucose and dehydroepiandrosterone sulfate in women with polycystic ovary syndrome but produced less consistent effects on insulin resistance and other hormonal outcomes [198,199]. Nevertheless, enhanced-bioavailability combinations require caution. Piperine can increase systemic curcumin exposure, and a reported case of severe hypoglycemia and transient loss of consciousness in a patient with previously undiagnosed insulinoma suggests that curcumin–piperine supplements may potentiate glucose lowering in individuals with abnormal insulin secretion [200]. Collectively, the current evidence supports the antidiabetic potential of *C. longa* through stimulation of insulin secretion, enhancement of insulin signaling and glucose uptake, inhibition of carbohydrate-digesting enzymes, preservation or regeneration of pancreatic β-cells, and attenuation of oxidative stress, inflammation, dyslipidemia, and obesity-associated metabolic dysfunction; however, clinical efficacy and safety should be interpreted according to the specific preparation and patient population. Nevertheless, the clinical evidence remains heterogeneous, with substantial variation in participant characteristics, baseline metabolic status, turmeric or curcumin formulations, doses, treatment durations, and concomitant therapies, while several studies involve relatively small sample sizes. Moreover, improvements in surrogate metabolic markers such as fasting glucose, HbA1c, HOMA-IR, or inflammatory biomarkers should not be interpreted as definitive evidence of reduced diabetic complications or long-term cardiovascular risk, particularly given the formulation-dependent bioavailability of curcumin and the limited availability of large, long-duration trials. These metabolic and anti-inflammatory effects are particularly relevant to hepatic protection, because insulin resistance, dyslipidemia, oxidative stress, and chronic inflammation are major drivers of fatty liver disease and progressive liver injury.

#### 2.4.7. Hepatoprotective Activity

Liver diseases, including acute hepatotoxicity, non-alcoholic fatty liver disease (NAFLD), non-alcoholic steatohepatitis (NASH), fibrosis, cirrhosis, and hepatocellular carcinoma, share interconnected pathological mechanisms involving oxidative stress, chronic inflammation, metabolic dysregulation, mitochondrial dysfunction, apoptosis, and extracellular matrix accumulation. Experimental studies indicate that *C. longa* and its principal constituent, curcumin, protect the liver by restoring endogenous antioxidant defenses, increasing glutathione, superoxide dismutase, catalase, and glutathione peroxidase activities, and reducing reactive oxygen species, malondialdehyde formation, and lipid peroxidation [201,202,203,204,205]. These effects are partly mediated through activation and nuclear translocation of nuclear factor erythroid 2-related factor 2 (Nrf2), which induces downstream antioxidant and cytoprotective proteins, including heme oxygenase-1 and glutathione-related enzymes [206]. Curcumin also attenuates inflammatory signaling by inhibiting M1 macrophage polarization, reducing hepatic macrophage infiltration, and suppressing the expression of TNF-α, IL-1β, IL-6, MCP-1, VCAM-1, CCR2, and other inflammatory mediators [207,208]. In alcohol-induced liver injury, turmeric extract further decreases CYP2E1-associated reactive oxygen species generation, thereby limiting oxidative damage, inflammatory cytokine production, and hepatocellular injury [209].

In metabolic and chronic liver disorders, *C. longa* reduces hepatic lipid accumulation by suppressing fatty acid uptake and de novo lipogenesis through downregulation of CD36, FATP2, FATP5, SREBP-1c, ACC, and FAS, while activating AMPK, PPARα, and CPT-1 to enhance mitochondrial fatty acid β-oxidation [210]. Curcumin also decreases cholesterol accumulation by inhibiting intestinal cholesterol absorption and hepatic cholesterol synthesis through modulation of NPC1L1, SREBP-2, and HMGCR, while restoration of endoplasmic reticulum redox balance alleviates unfolded-protein stress and lipid dysregulation [211,212]. Its anti-fibrotic activity is associated with inhibition of hepatic stellate-cell activation and reductions in TGF-β, α-SMA, collagen type I, PDGFRβ, and TIMP-1, thereby limiting extracellular matrix deposition and fibrosis progression [213]. In parallel, regulation of autophagy- and apoptosis-related proteins, including LC3-II, SQSTM1, and Bcl-2, may preserve hepatocyte viability and delay progression toward hepatocellular carcinoma [214]. Consistent with these mechanisms, a randomized placebo-controlled trial in patients with NAFLD demonstrated that curcumin supplementation reduced hepatic fat content, serum ALT and AST, fasting glucose, glycated hemoglobin, triglycerides, total cholesterol, low-density lipoprotein cholesterol, and body mass index [215]. Nevertheless, larger multicenter trials are required to establish optimal dosage, long-term safety, and clinically effective formulations capable of overcoming the limited oral bioavailability of native curcumin. However, much of the mechanistic evidence remains derived from cellular and animal models, and the doses producing hepatoprotective effects experimentally may not directly correspond to clinically achievable systemic exposure in humans. In addition, current clinical studies rely largely on surrogate outcomes such as liver enzymes, metabolic parameters, and hepatic fat content, while evidence for long-term effects on fibrosis progression, cirrhosis, hepatocellular carcinoma, or liver-related clinical outcomes remains limited and may be influenced by differences in curcumin formulation and bioavailability. More broadly, the combined regulation of oxidative stress, inflammation, cellular survival, and tissue remodeling also underlies the reported wound-healing effects of curcumin and turmeric-derived preparations.

#### 2.4.8. Wound Healing Activity

Wound healing is a highly coordinated biological process involving hemostasis, inflammation, proliferation, angiogenesis, extracellular matrix deposition, re-epithelialization, and tissue remodeling. Curcumin, the principal bioactive constituent of *C. longa*, promotes wound repair through the simultaneous regulation of several molecular and cellular pathways rather than through a single pharmacological target. During the inflammatory phase, curcumin helps restore inflammatory homeostasis by modulating TNF-α expression and suppressing excessive activation of NF-κB, MCP-1, and MMP-9, thereby limiting prolonged leukocyte recruitment, inflammatory tissue damage, and extracellular matrix degradation [216,217,218]. Importantly, curcumin does not appear to completely inhibit the early inflammatory response; instead, it facilitates the timely transition from inflammation to proliferation, which is essential for effective tissue regeneration [218]. Its polyphenolic structure also enables direct and indirect scavenging of reactive oxygen species, protecting fibroblasts, keratinocytes, and endothelial cells from oxidative injury while preserving collagen synthesis and cellular viability [217,219]. During the proliferative phase, curcumin enhances fibroblast migration and proliferation through activation of Wnt/β-catenin-related signaling and inhibition of GSK3β, leading to increased granulation tissue formation and extracellular matrix production [216,220]. It further promotes fibroblast-to-myofibroblast differentiation, as reflected by increased α-smooth muscle actin expression, thereby strengthening wound contraction and facilitating wound closure [218].

Curcumin also supports angiogenesis by increasing the formation of CD31-positive microvessels and improving oxygen and nutrient delivery to regenerating tissues, an effect that is particularly important in diabetic wounds characterized by impaired vascularization [97,217,221]. Concurrently, it stimulates collagen deposition, improves collagen fiber organization, limits excessive matrix breakdown through MMP-9 suppression, and accelerates re-epithelialization and restoration of normal skin architecture [97,217,221,222]. Its broad-spectrum antimicrobial activity against Gram-positive and Gram-negative bacteria further reduces microbial burden and prevents infection-associated delays in healing [219,221,223,224]. These effects can be enhanced by combining curcumin with complementary materials or bioactive agents, including chitosan, zinc oxide nanoparticles, epidermal growth factor, and mesenchymal stem cells, which may provide synergistic antioxidant, antimicrobial, angiogenic, and regenerative benefits [219,223,225]. Collectively, these findings indicate that curcumin promotes wound healing through coordinated control of inflammation, oxidative stress, fibroblast function, angiogenesis, matrix remodeling, wound contraction, and microbial defense. Despite these promising pharmacological activities, the clinical application of curcumin is constrained by poor aqueous solubility, chemical instability, limited skin penetration, and low bioavailability. Accordingly, advanced delivery systems, including nanomicelles, nanoemulsions, nanofibers, hydrogels, nanoemulgels, ethosomes, and multifunctional composite dressings, have been developed to improve curcumin stability, local retention, sustained release, and therapeutic concentrations at the wound site. Hydrogel-based platforms are particularly advantageous because they maintain a moist environment, absorb exudates, provide mechanical protection, and facilitate cellular infiltration while delivering curcumin in a controlled manner [97,217,219,221]. Overall, substantial preclinical evidence supports curcumin as a multifunctional wound-healing agent; however, future studies should prioritize standardized formulations, pharmacokinetic characterization, long-term safety assessment, and well-designed randomized clinical trials to support its translation into routine clinical practice. However, most evidence supporting the wound-healing effects of curcumin remains derived from in vitro studies and animal wound models, which may not fully reproduce the complexity of chronic human wounds, particularly those associated with diabetes, vascular insufficiency, or infection. Human evidence remains comparatively limited and heterogeneous with respect to formulation, wound type, treatment duration, and clinical endpoints; therefore, improvements in wound closure or histological parameters observed preclinically should not yet be interpreted as definitive clinical efficacy.

Across the pharmacological activities reviewed, the strength of evidence remains substantially greater for rhizome-derived preparations, curcumin, and curcuminoids than for non-rhizome tissues. Evidence for leaf, flower, root, and root tuber is comparatively limited and is largely derived from in vitro or preliminary preclinical studies, with few standardized head-to-head comparisons among plant organs. Consequently, the biological potential of non-rhizome tissues should be regarded as promising but insufficiently established, and future studies should determine whether these organs provide distinct or complementary activities rather than assuming equivalence with the rhizome.

#### 2.4.9. Safety, Drug Interactions, and Precautions

Turmeric and curcumin are generally well tolerated in human studies, although their safety profile depends on dose, formulation, duration of administration, and individual susceptibility. Dose-escalation and phase I studies have reported acceptable short-term tolerability of curcumin at oral doses up to 8–12 g/day, with adverse events consisting mainly of mild gastrointestinal symptoms [226,227]. However, clinically important hepatotoxicity has increasingly been recognized. Data from the U.S. Drug-Induced Liver Injury Network identified turmeric-associated liver injury with a predominantly hepatocellular pattern, and HLA-B*35:01 was frequently observed among affected individuals, suggesting a possible genetic susceptibility [228]. These findings indicate that the safety of concentrated turmeric or curcumin supplements should be distinguished from conventional dietary use. In addition, human studies have demonstrated dose-dependent gallbladder contraction following curcumin administration, supporting caution in individuals with gallstones, bile-duct obstruction, or other biliary disorders [229,230]. Evidence regarding long-term use, pregnancy and lactation, and newer highly bioavailable formulations remains comparatively limited.

Potential herb–drug interactions should also be considered, particularly for concentrated curcumin preparations and formulations containing bioavailability enhancers such as piperine. In healthy volunteers, curcumin significantly increased systemic exposure to sulfasalazine, supporting inhibition of intestinal breast cancer resistance protein (BCRP/ABCG2) [231]. In contrast, a randomized crossover study found no clinically meaningful short-term effects of a curcuminoid–piperine formulation on the pharmacokinetics of midazolam, flurbiprofen, or paracetamol, indicating that interaction risk may depend on the metabolic pathway, dose, formulation, and duration of exposure [232]. Case reports have also described elevated tacrolimus concentrations and nephrotoxicity associated with high turmeric intake, although subsequent clinical observations have not consistently reproduced this interaction [233,234]. Mechanistic studies further indicate that curcuminoids and piperine can modulate CYP3A4, CYP2C9, P-glycoprotein, UGT, and related drug-metabolizing pathways [235,236]. Accordingly, caution is warranted when turmeric or curcumin supplements are used concomitantly with drugs having a narrow therapeutic index, including immunosuppressants and anticoagulant or antiplatelet therapies, particularly when highly concentrated or bioavailability-enhanced formulations are involved.

### 2.5. Whole-Plant Valorization and Bioeconomy Potential

Beyond its established pharmacological and commercial importance, *C. longa* represents a promising renewable biomass resource for sustainable industrial development. Conventional turmeric production primarily focuses on the rhizome, whereas leaf, pseudostem, inflorescence, root, and residues generated during curcuminoid, pigment, juice, and essential-oil processing are frequently discarded or underutilized. A whole-plant biorefinery approach could convert these materials into multiple product streams through sequential or cascading extraction, thereby maximizing the value recovered from each unit of biomass. Within such a system, the rhizome may initially be processed for curcuminoids, oleoresins, natural pigments, and turmerone-rich essential oils, while the remaining carbohydrate-rich matrix can subsequently be utilized as a source of starch, dietary fiber, fermentable sugars, and biomaterials [237,238,239]. Turmeric-processing residues have also been investigated as substrates for the production of lactic acid and ethanol, demonstrating that biomass remaining after extraction retains considerable potential for further biotechnological conversion rather than disposal [237,240].

The aerial parts provide additional opportunities for product diversification. Turmeric leaf contains volatile terpenoids, phenolic compounds, cellulose, and other structural polysaccharides that may be recovered for use in natural preservatives, botanical pesticides, active packaging, fibers, and biodegradable materials. In particular, leaf essential oils exhibit antimicrobial, antioxidant, antifungal, and insect-repellent properties, supporting their potential application in food protection and crop-management products [39]. Cellulose fibers isolated from turmeric pseudostems and other aerial residues have likewise been proposed as renewable reinforcing materials, while starch–fiber fractions obtained from rhizome-processing residues can be modified to produce biodegradable films and related biopolymer products [240,241]. Flower and inflorescence, although generated in smaller quantities than leaf and rhizome residues, may provide phenolic-rich extracts, pigments, aromatic constituents, or reducing agents for green synthesis applications. After the recovery of higher-value constituents, the remaining lignocellulosic biomass may be directed toward composting, organic fertilizers, anaerobic digestion, bioenergy generation, biochar production, or other soil-improvement products, thus creating a cascading utilization pathway in which material and energy recovery are prioritized before final disposal.

Quantitative evidence supporting turmeric biorefinery remains limited but is beginning to emerge. For non-rhizome biomass, hydro-distillation of fresh *C. longa* leaf yielded 1.62 ± 0.34% (*v*/*w*) essential oil, indicating their potential as an additional source of volatile compounds [39]. At pilot scale, steam distillation of fresh turmeric rhizome under optimized conditions yielded 0.911% essential oil, with 88.24% oil recovery and 92.72% energy utilization; the economic assessment further indicated an internal rate of return of 26.68% and a payback period of 2.78 years [242]. Techno-economic modelling of an integrated supercritical-fluid extraction, pressurized-liquid extraction, and supercritical antisolvent process showed that increasing processing capacity from 2 × 50 L to 2 × 500 L reduced the manufacturing cost of turmeric volatile oil from US$112.70 to US$85.58 kg^−1^ and that of a curcuminoid-rich powdered extract from US$174.80 to US$141.63 kg^−1^ [243]. However, these quantitative assessments remain largely rhizome-centered, while comparable techno-economic, energy-balance, life cycle, and commercial-scale data for leaf, flower, root, and pseudostem are still scarce. At present, the economic competitiveness of leaf- and flower-derived extraction relative to rhizome-based production cannot be established because direct comparative techno-economic data are still lacking. Thus, whole-plant turmeric biorefinery should currently be regarded as a promising concept requiring further validation through pilot-scale studies, techno-economic analysis, and comprehensive life-cycle assessment. Despite these promising findings, important scalability barriers remain before whole-plant turmeric biorefinery can be commercially implemented. Variability in biomass availability, moisture, composition, and seasonal supply may complicate feedstock standardization, storage, transport, and continuous processing, while integrated biorefineries also face challenges related to capital investment, process integration, and market uncertainty [244,245]. In addition, some green extraction technologies, including natural deep eutectic solvents, may be limited by high viscosity, solvent recovery, and recyclability issues at larger scales [246]. Thus, laboratory-scale extraction performance alone is insufficient to establish industrial feasibility, and further pilot-scale, techno-economic, and life-cycle assessments are required.

The implementation of this integrated model would support circular-bioeconomy principles by reducing agricultural waste, diversifying farmer and processor income, and encouraging the establishment of decentralized value chains in turmeric-producing regions. Nevertheless, successful industrial translation requires optimization of biomass collection and storage, tissue-specific extraction procedures, solvent and water recycling, product standardization, and the management of seasonal and chemotypic variation. Green technologies—including supercritical-fluid extraction, pressurized-liquid extraction, ultrasound- and microwave-assisted extraction, natural deep eutectic solvents, enzymatic pretreatment, and solid-state fermentation—may improve compound recovery while reducing solvent consumption and processing time [247,248]. Metabolomics and chemometric analysis can further support the identification and prioritization of valuable compounds across different plant fractions, whereas techno-economic analysis and life-cycle assessment are necessary to determine whether proposed processing chains provide genuine environmental and economic advantages at scale [249,250,251]. By integrating traditional knowledge, modern phytochemical technologies, and waste-to-value conversion, *C. longa* may provide a useful model for near-zero-waste utilization and sustainable value-added production, provided that its technical scalability, economic feasibility, and environmental performance are further validated.

### 2.6. Industrial Applications and Commercial Utilization of C. longa

The rhizomes of *C. longa* are widely used in the food industry as spices, natural colorants, flavoring agents, preservatives, and functional ingredients. Curcuminoids, particularly curcumin, provide the characteristic yellow–orange color of curries, sauces, dairy products, confectionery, and beverages, while their antioxidant and antimicrobial properties may improve product stability and shelf life [4,252,253]. Turmeric extracts, oleoresins, and essential oils are also being explored as natural preservatives and components of active packaging. In addition to rhizome, the leaf and flower contain phenolic compounds, flavonoids, and volatile constituents with antioxidant potential, supporting their possible use in herbal beverages, fragrances, and other value-added products [9,89]. However, the composition and biological properties of these materials can vary according to genotype, geographical origin, cultivation conditions, developmental stage, and extraction method.

In pharmaceutical and nutraceutical applications, turmeric and curcuminoid-rich extracts are commonly formulated as capsules, tablets, soft gels, syrups, creams, gels, and wound-care products. Curcumin has been investigated for its antioxidant, anti-inflammatory, antimicrobial, immunomodulatory, and wound-healing activities, with potential applications in inflammatory diseases, osteoarthritis, metabolic disorders, neurodegenerative conditions, and supportive cancer care [253,254]. Nevertheless, its low aqueous solubility, poor intestinal absorption, rapid metabolism, and limited systemic bioavailability remain major challenges [255]. Consequently, advanced delivery systems, including phospholipid complexes, liposomes, polymeric nanoparticles, solid lipid nanoparticles, nanoemulsions, micelles, and self-emulsifying formulations, have been developed to improve curcumin stability, absorption, and controlled delivery [254,256,257]. Turmeric volatile oils, particularly those rich in ar-turmerone, α-turmerone, and β-turmerone, have also attracted interest for their anti-inflammatory, antimicrobial, and neuroprotective properties, although most supporting evidence remains preclinical [58].

Turmeric-derived ingredients are further incorporated into facial creams, serums, soaps, shampoos, sunscreens, anti-aging products, and formulations intended to reduce hyperpigmentation or improve uneven skin tone. These applications are supported by their antioxidant, anti-inflammatory, antimicrobial, photoprotective, and wound-healing properties [1,258,259,260]. Underutilized plant parts and processing residues are also being explored as additional commercial feedstocks. Leaf, flower, spent rhizome residues, and extraction by-products may provide essential oils, natural dyes, dietary fiber, and biopolymer additives, while some residues may be further directed toward compost or bioenergy production. These applications are discussed here primarily from a product-development and utilization perspective, whereas their resource-efficiency, environmental, and sustainability implications are considered separately in Section 2.6. Beyond *C. longa*, ornamental species of the genus *Curcuma*, particularly *C. alismatifolia*, are commercially important as potted plants, cut flowers, and landscaping species because of their attractive inflorescences and relatively long vase life [261,262].

### 2.7. Sustainability and SDG Implications of Whole-Plant C. longa Utilization

Whereas Section 2.5 focuses on established and emerging product applications, this section considers whether whole-plant utilization can provide broader sustainability benefits in terms of resource efficiency, waste reduction, environmental performance, and socioeconomic value. Beyond its established pharmacological and commercial significance, *C. longa* represents a promising biomass resource whose sustainability potential derives from the morphological and phytochemical diversity of the whole plant. Whereas the rhizome is extensively exploited as a source of curcuminoids, volatile oils, natural pigments, flavoring agents, and functional ingredients, the leaf, pseudostem, flower, root, and post-extraction residues may provide additional essential oils, phenolic compounds, polysaccharides, cellulose-rich fibers, and other structurally or biologically valuable materials [4,37,39,241,254]. The integration of these tissue-specific chemical constituents and biological properties into a whole-plant biorefinery could enable the sequential recovery of high-value compounds, followed by the conversion of residual biomass into agricultural, material, or bio-based industrial products. Such cascading utilization is consistent with circular bioeconomy principles because it improves resource efficiency, reduces the disposal of agricultural residues, and broadens the economic value obtained from each cultivated plant. Accordingly, the integration of medicinal, nutritional, ornamental, and industrial applications of *C. longa* may contribute particularly to SDG 3 through the development of evidence-based health products, SDG 8 through rural employment and value creation, SDG 9 through bioprocessing and product innovation, and SDG 12 through waste prevention and responsible biomass utilization [36,263].

Nevertheless, the sustainability of *C. longa*-based production cannot be evaluated solely by the number of products obtainable from the plant. Its long-term development requires coordinated attention to product efficacy, compositional consistency, safety, regulatory oversight, and environmental performance. Curcumin continues to exhibit limited oral availability due to its low aqueous solubility, restricted absorption, rapid metabolism, and rapid systemic elimination. These challenges have led to the widespread development of piperine-containing products and advanced delivery systems intended to enhance exposure [253,255]. However, increased systemic availability may also alter the safety profile of curcumin preparations, particularly when concentrated extracts or absorption-enhancing formulations are consumed without formulation-specific dosage guidance. A recent assessment of 125 commercially available turmeric supplements identified substantial differences in curcuminoid disclosure, recommended intake, inclusion of bioavailability-enhancing substances, safety warnings, and regulatory practices across five countries; approximately one-third of the evaluated products did not disclose their curcuminoid content. The study also emphasized the need for improved labeling, standardized formulations, long-term clinical safety evaluation, and clearer dosage recommendations, particularly for highly bioavailable products [264]. Reports of liver injury associated with turmeric or curcumin supplements further indicate that the safety of concentrated preparations should be distinguished from that of turmeric used conventionally as a food ingredient, although causality, individual susceptibility, dosage, co-ingredients, and formulation characteristics require careful interpretation [264]. From an environmental perspective, proposed benefits of whole-plant utilization should also be demonstrated rather than assumed. The environmental advantages proposed for whole-plant utilization should be verified through life-cycle assessment, material and energy balances, cultivation-input analysis, and techno-economic evaluation rather than assumed solely from the renewable nature of the biomass [265]. Although rhizome and, to a lesser extent, leaf and pseudostem have received increasing scientific and industrial attention, the flower and other morphologically distinct organs, including root tuber, remain comparatively underutilized and insufficiently characterized; consequently, further phytochemical, biological, technological, safety, and sustainability-oriented studies of these plant parts are warranted.

## 3. Future Perspectives

Future research on *C. longa* should move beyond the traditional emphasis on rhizome and curcuminoids toward a more systematic evaluation of the whole plant, including leaf, flower, root, root tuber, pseudostem, and processing residues. Greater attention is needed to organ-specific phytochemical profiles, standardized extraction procedures, safety assessment, and biological activities to determine whether underutilized tissues can provide reproducible and commercially relevant sources of functional compounds. At the clinical level, stronger evidence will require well-designed trials using chemically standardized preparations, clearly defined participant populations, appropriate intervention durations, and formulations with characterized bioavailability. These factors are particularly important because differences in extract composition, dose, formulation, disease status, and study design likely contribute to the heterogeneous outcomes reported among existing clinical studies. Future investigations should therefore integrate phytochemical characterization, pharmacokinetics, mechanistic biomarkers, and clinical endpoints to better establish relationships between turmeric preparations and therapeutic responses. Future research should particularly prioritize flower and root tuber through organ-specific metabolomic profiling, standardized bioactivity testing, and direct comparison with rhizome and leaf. For floral tissues, studies should also examine flowering stage, seasonal biomass availability, harvest yield, and post-harvest stability to determine whether their phytochemical and biological value can be translated into a reliable raw-material supply.

The translation of whole-plant utilization into a circular bioeconomy will also require greater emphasis on technological feasibility, environmental performance, and regulatory acceptance. Although laboratory-scale studies demonstrate opportunities to convert turmeric leaf, pseudostem, root, and processing residues into essential oils, natural preservatives, fibers, biodegradable materials, fermentation substrates, and bioenergy, challenges remain in biomass collection, seasonal variability, extraction efficiency, process scale-up, product standardization, cost-effectiveness, and life-cycle sustainability. Regulatory frameworks represent an additional translational consideration because current official recognition predominantly focuses on the rhizome. The Thai Herbal Pharmacopoeia and the European Medicines Agency monograph specifically recognize *C. longa* rhizome [26,266], while the US FDA considers the botanical part used as an important element of dietary-ingredient identity and may require additional assessment for a different plant part [267]. ASEAN guidelines similarly require clear identification of the botanical material and plant part used, whereas non-rhizome materials intended as foods in the European Union may require evaluation under the Novel Food framework when sufficient history of consumption cannot be demonstrated [268]. Consequently, future commercialization of non-rhizome turmeric resources should be supported by organ-specific evidence of identity, composition, quality, safety, efficacy where relevant, scalability, and environmental benefit. Addressing these scientific, technological, economic, and regulatory gaps will be essential for translating the whole-plant concept from laboratory-scale potential into practical and sustainable utilization.

## 4. Materials and Methods

A comprehensive literature review was conducted to compile information on the botany, traditional uses, phytochemistry, pharmacological activities, industrial applications, and sustainable utilization of *C. longa* L. The literature search was performed from January to August 2026 using Google Scholar, PubMed, ScienceDirect, Scopus, and Web of Science. Relevant publications were identified using individual and combined keywords, including “Curcuma longa”, “turmeric”, “taxonomy”, “ethnobotany”, “phytochemistry”, “curcumin”, “curcuminoids”, “essential oil”, “pharmacological activity”, “industrial applications”, “biomass”, “biorefinery”, “waste valorization”, and “circular bioeconomy”. Additional studies were identified through citation tracking and cross-referencing of retrieved publications. Authoritative botanical and taxonomic information was also consulted from recognized sources, including the Flora of Thailand, World Flora Online, and Plants of the World Online.

Peer-reviewed English-language original articles, clinical and preclinical studies, relevant reviews, pharmacopoeial sources, and authoritative botanical references were considered for inclusion. Conference abstracts, duplicate publications, studies with insufficient methodological information, and reports outside the scope of the review were excluded. Clinical studies were included when they evaluated *C. longa*, turmeric extracts, curcuminoids, or defined turmeric-derived preparations and reported relevant clinical, safety, pharmacokinetic, or biomarker outcomes. For each eligible study, relevant information was extracted on the plant material or preparation, chemical composition where available, experimental or clinical model, dose or concentration, intervention duration, and principal outcomes. Particular attention was given to rhizome and underutilized plant parts, including leaf, flower, root, root tuber, pseudostem, and processing residues. The evidence was narratively synthesized according to botanical characteristics, chemical classes, pharmacological activities, industrial applications, and whole-plant valorization, with emphasis on quantitative findings and experimentally supported mechanisms where available. No systematic review protocol or PRISMA-based methodology was applied because the objective was to provide a broad narrative synthesis across heterogeneous botanical, pharmacological, clinical, and sustainability evidence. The methodological quality of clinical evidence was therefore assessed qualitatively during synthesis rather than through a formal systematic risk-of-bias tool.

## 5. Conclusions

*C. longa* is a multifunctional medicinal and economic plant whose value extends well beyond the traditionally exploited rhizome. Research has historically focused on the rhizome and curcuminoids; however, this review integrates previously scattered information across leaf, flower, root, root tuber, pseudostem, and processing residues to provide a broader whole-plant perspective. Curcuminoids and turmerone-rich essential oils remain the best-characterized constituents, while other plant organs also contain diverse bioactive metabolites and represent underutilized resources. The accumulated evidence, particularly from in vitro and animal studies, indicates a broad spectrum of antioxidant, anti-inflammatory, antimicrobial, antiviral, anticancer, antidiabetic, hepatoprotective, and wound-healing effects associated with multiple molecular targets and signaling pathways. However, the strength of evidence varies considerably among these activities, and many findings remain predominantly preclinical; therefore, therapeutic efficacy in humans cannot yet be inferred consistently across indications. Further studies using standardized preparations, appropriately characterized formulations, well-designed clinical trials, and formulation-specific safety evaluation are required. From a sustainability perspective, the integration of underutilized plant parts and processing residues into cascading biorefinery systems could expand the production of essential oils, natural preservatives, fibers, biodegradable materials, fermentation products, and bioenergy while reducing agricultural waste. Nevertheless, evidence supporting the industrial translation of whole-plant utilization remains uneven, particularly for non-rhizome tissues, and further validation of scalability, economic feasibility, environmental performance, and regulatory acceptance is required. Future research should therefore prioritize tissue-specific phytochemical characterization, especially of flower and root tuber, together with green extraction technologies, product standardization, techno-economic analysis, and life-cycle assessment. By consolidating fragmented evidence across different plant organs and identifying these remaining knowledge gaps, this review provides a framework for future research toward more evidence-based and sustainable whole-plant utilization of *C. longa*.

## Figures and Tables

**Table 1:** Major phytochemical constituents identified from different parts of *C. longa*.

Group	Name	CAS Number	Plant Parts	References
Curcuminoids and diarylheptanoids Compound	1,5-bis(4-hydroxy-3-methoxyphenyl)-penta-(1E,4E)-1,4-dien-3-one	NR	root tuber	[40]
	1,5-dihydroxy-1,7-bis(4-hydroxy-3-methoxyphenyl)-4,6-heptadiene-3-one	NR	rhizome	[41]
	1,5-dihydroxy-1,7-bis(4-hydroxyphenyl)-4,6-heptadiene-3-one	NR	rhizome	[41]
	1,5-dihydroxy-1-(4-hydroxy-3-methoxyphenyl)-7-(4-hydroxyphenyl)-4,6-heptadiene-3-one	NR	rhizome	[41]
	1,5-dihydroxy-1-(4-hydroxyphenyl)-7-(4-hydroxy-3-methoxyphenyl)-4,6-heptadiene-3-one	NR	rhizome	[41]
	1,7-bis(4-hydroxyphenyl)-1-heptene-3,5-dione	NR	rhizome	[42]
	1-(4-hydroxy-3-methoxyphenyl)-7-(3,4-dihydroxyphenyl)-1,6-heptadiene-3, 5-dione	NR	rhizome	[41]
	1-(4-hydroxy-3-methoxyphenyl)-5-(4-hydroxyphenyl)-1,4-pentadiene-3-one	NR	rhizome	[41]
	3-hydroxy-1,7-bis-(4-hydroxyphenyl)-6- heptene-1,5-dione	NR	rhizome	[41]
	5-hydroxyl-1-(4-hydroxy-3-methoxyphenyl)-7-(4-hydroxyphenyl)-4,6-heptadiene-3-one	NR	rhizome	[42]
	5-hydroxyl-1,7-bis(4-hydroxy-3-methoxyphenyl)-4,6-heptadiene-3-one	NR	rhizome	[42]
	5-hydroxyl-7-(4-hydroxy-3-methoxyphenyl)-1-(4-hydroxyphenyl)-4,6-heptadiene-3-one	NR	rhizome	[42]
	bisdemethoxycurcumin	33171-05-0	rhizome	[43,44,45]
	curcumin	458-37-7	rhizome	[44,45,46,47]
	cyclocurcumin	153127-42-5	rhizome	[48,49]
	demethoxycurcumin	22608-11-3	rhizome	[44,45,46]
	methylcurcumin	NR	rhizome	[46]
	monodemethylcurcumin	149732-51-4	rhizome	[45]
	octahydrocurcumin	36062-07-4	leaf	[50]
	sodium curcuminate	NR	rhizome	[46]
	tetrahydroxycurcumin	36062-04-1	rhizome	[51]
Other phenolic compounds and phenylpropanoid derivatives	(E)-ferulic acid	537-98-4	rhizome	[52]
	(Z)-ferulic acid	1014-83-1	rhizome	[52]
	2-methoxy-4-vinylphenol	7786-61-0	rhizome	[53]
	4-hydroxycinnamic acid	501-98-4	rhizome	[45]
	calebin A	336784-82-8	rhizome	[44]
	dehydrozingerone	1080-12-2	root, rhizome	[49,52]
	eugenol	97-53-0	leaf, rhizome	[53,54,55]
	vanillic acid	121-34-6	rhizome	[52]
	vanillin	121-33-5	rhizome	[45,52]
	zingerone	122-48-5	root tuber	[56]
Monoterpenoids	(−)-terpinen-4-ol	20126-76-5	rhizome, root tuber	[57]
	(E)-β-ocimene	3779-61-1	rhizome	[58]
	(Z)-β-ocimene (cis-ocimene)	3338-55-4	rhizome	[59,60]
	1,8-cineol (eucalyptol)	470-82-6	leaf, rhizome, root tuber	[53,55,58,60]
	2-isopropylidene-3-methylhexa-3,5-dienal	NR	rhizome	[53]
	2-menthen-1-ol	NR	leaf	[50]
	3,7,7-trimethyl-1,3,5-cycloheptatriene	3479-89-8	rhizome	[61]
	3-carene	13466-78-9	rhizome, root tuber	[57]
	4-isopropenyl-1,2-dimethylcyclohex-ane-2-enol	NR	rhizome	[53]
	α-fenchene	471-84-1	flower, leaf	[9]
	α-phellantrene	99-83-2	leaf, rhizome, root tuber	[57,60]
	α-pinene	80-56-8	leaf, rhizome	[54,59]
	α-terpinene	99-86-5	rhizome, root tuber	[57]
	α-terpineol	98-55-5	rhizome, root tuber	[57]
	α-thujone	546-80-5	rhizome	[53]
	β-pinene	127-91-3	flower, leaf, rhizome, root tuber	[9,57,59,62]
	borneol	507-70-0	rhizome	[59]
	camphene	79-92-5	rhizome, root tuber	[57]
	camphor	76-22-2	rhizome	[62]
	cis-*p*-menth-2,8-dienol	NR	rhizome	[53]
	cis-*p*-mentha-1(7),8-dien-2-ol	22626-43-3	rhizome	[61]
	cis-sabinol	471-16-9	rhizome	[53]
	D-limonene	5989-27-5	rhizome, root tuber	[57]
	isopulegol	89-79-2	leaf	[50]
	limonene	138-86-3	leaf	[54]
	linalool	78-70-6	rhizome	[59]
	m-cymene	535-77-3	rhizome, root tuber	[57]
	menth-1-en-9-ol	18479-68-0	leaf	[50]
	myrcene	123-35-3	rhizome, leaf, flowers	[9,54]
	*o*-cymene	527-84-4	rhizome	[53]
	*p*-cymen-8-ol	1197-01-9	rhizome, root tuber	[57]
	*p*-cymene	99-87-6	leaf, rhizome	[54,60]
	sabinene	3387-41-5	flower, leaf	[9]
	terpinolene	586-62-9	flower, leaf, rhizome	[54,60,63]
	trans-chrysanthenyl acetate	NR	rhizome	[53]
	tricyclene	508-32-7	leaf, flower	[9]
Sesquiterpenoids	(−)-β-sesquiphellandrene	20307-84-0	rhizome, root tuber	[57]
	(E)-α-santalal	19903-70-9	rhizome	[62]
	(E)-atlantone	108645-54-1	rhizome	[62]
	(E)-β-farnesene	18794-84-8	rhizome, root tuber	[57,61,62]
	(E)-γ-atlantone	108549-47-9	rhizome	[62]
	7-epi-sesquithujene	159407-35-9	rhizome, root tuber	[57,62]
	8,12-epoxygermacra-1(10),4,7,11-tetraene	NR	leaf	[50]
	α-acorenol	28296-85-7	rhizome	[62]
	α-atlantone	26294-59-7	rhizome	[64]
	α-cedrene	469-61-4	rhizome	[53]
	α-humulene	6753-98-6	rhizome, root tuber	[57,62]
	α-santalene	512-61-8	rhizome	[32]
	α-turmerone	532-65-0	rhizome, root tuber	[32,60,65]
	α-zingiberene	495-60-3	rhizome, root tuber	[55,56,62]
	ar-curcumene	644-30-4	rhizome, root tuber	[32,53,62,65]
	ar-turmerol	38142-57-3	rhizome, root tuber	[62,66]
	ar-turmerone	532-65-0	rhizome, root tuber	[32,45,46,53,55,57,60,61,62,64,67,68]
	β-bisabolene	495-61-4	rhizome, root tuber	[53,57,61,62]
	β-caryophyllene	87-44-5	rhizome, root tuber	[53,55,56]
	β-cedrene	546-28-1	rhizome	[53,62]
	β-curcumene	28976-67-2	rhizome	[69]
	β-elemene	515-13-9	rhizome	[53,62]
	β-selinene	17066-67-0	rhizome	[62]
	β-sesquiphellandrene	20307-83-9	rhizome, root tuber	[53,55,57,62,63]
	β-turmerone	82508-14-3	rhizome, root tuber	[32,53,55,57,60,62]
	β-vatirenene	27840-40-0	rhizome	[53]
	β-ylangene	20479-06-5	rhizome	[62]
	bisabolone	66964-98-5	rhizome	[62]
	bisacurone	120681-68-1	rhizome, root tuber	[56,70]
	bisacurone A	NR	rhizome, root tuber	[56,70]
	bisacurone B	NR	rhizome, root tuber	[56,70]
	caryophyllene oxide	1139-30-6	rhizome, root tuber	[57,62]
	cis-α-bergamotene	18252-46-5	rhizome, root tuber	[57,62]
	cis-sesquisabinene hydrate	58319-05-4	rhizome	[62]
	cryptomeridiol	4666-84-6	rhizome	[62]
	curcumenol	19431-84-6	rhizome	[53,62]
	curcuphenol	69301-27-5	rhizome, root tuber	[57]
	curdione	13657-68-6	rhizome	[62]
	δ-cadinene	483-76-1	rhizome	[62]
	epicurzerenone	20085-85-2	rhizome	[62]
	farnesol	4602-84-0	leaf	[54]
	furanodienone (8,12-epoxygermacra-1(10), 4,7,11-tetraen-6-one	24268-41-5	leaf	[50]
	γ-curcumene	28976-68-3	rhizome, root tuber	[57]
	germacrene B	15423-57-1	rhizome	[62]
	germacron-13-al	NR	rhizome	[71]
	germacrone	6902-91-6	rhizome	[62]
	nerolidol	7212-44-4	rhizome, root tuber	[57]
	sesquisabinene	58319-04-3	rhizome	[61]
	trans-α-bergamotene	13474-59-4	rhizome	[61]
	trans-nuciferol	39599-18-3	rhizome	[62]
	turmerol	NR	rhizome	[64]
	turmerone Q	NR	rhizome	[70]
	turmeronol A	NR	rhizome, root tuber	[40,45,56,72]
	turmeronol B	131651-38-2	rhizome	[72]
	zingiberenol	58334-55-7	rhizome, root tuber	[57,62]
Diterpenoids	(E)-labda-8(17),12-dien-15,16-dial	104263-85-6	leaf	[73]
	(E,E,E)-3,7,11,15- tetramethylhexadeca-1,3,6,10,14-pentaene	77898-97-6	rhizome	[74]
	(E,E)- 3,7,11,15-tetramethylhexadeca- 1,6,10,14-tetraen-3-ol	NR	rhizome	[74]
	2,6,11,15-tetramethyl-hexadeca-2,6,8,10,14-pentaene	NR	rhizome	[74]
	coronadiene	NR	leaf	[50]
	geranyl-p-cumene	NR	rhizome	[53]
	phytol	150-86-7	rhizome	[75]
Fatty acids and lipid derivatives	8,11-octadecadienoic acid, methyl ester	NR	rhizome	[75]
	α-linolenic acid	463-40-1	rhizome, root tuber	[76]
	arachidic acid	506-30-9	rhizome, root tuber	[76]
	docosanoic acid	112-85-6	rhizome, root tuber	[76]
	heptadecanoic acid	506-12-7	rhizome, root tuber	[76]
	lignoceric acid	557-59-5	rhizome, root tuber	[76]
	linoleic acid	60-33-3	rhizome, root tuber	[75,76]
	oleic acid	112-80-1	rhizome, root tuber	[61,75,76]
	palmitic acid	57-10-3	rhizome, root tuber	[61,75]
	palmitoleic acid	373-49-9	root tuber	[76]
	pentadecanoic acid	1002-84-2	rhizome, root tuber	[76]
	stearic acid	57-11-4	rhizome, root tuber	[61,75,76]
Sterols and steroidal compound	20-oxopregn-16-en-12-yl acetate	NR	rhizome	[52]
	β-sitosterol	83-46-5	rhizome	[52]
	gitoxigenin	545-20-4	rhizome	[52]
	stigmasterol	83-48-7	rhizome	[52]
Triterpenoids	hop-17(21)-en-3-ol	NR	rhizome	[77]
	hop-17(21)-en-3-yl acetate	NR	rhizome	[77]
	hopenone I	NR	rhizome	[77]
Alkaloids and other nitrogen-containing compounds	2,3,5,6-tetramethylpyrazine	1124-11-4	rhizome, root tuber	[57]
	2-(2′-methyl-1′-propenyl)-4,6-dimethyl-7-hydroxyquinoline	NR	root tuber	[56]
Other compounds	2-tridecanol	1653-31-2	rhizome	[62]
	2-undecanone	112-12-9	rhizome	[62]
	3,3,5,5-tetramethylcyclopentene	38667-10-6	rhizome, root tuber	[57,62]
	hemellitol	526-73-8	rhizome	[53]
	phthalic acid	88-99-3	rhizome	[61]
	undecanol	112-42-5	leaf	[60]

NR, CAS Registry Number, could not be reliably identified from the cited literature or authoritative publicly accessible chemical databases. Because the compounds listed in Table 1 were reported using heterogeneous analytical approaches and different levels of structural confirmation, detailed information on the plant part, identification method, level of structural confirmation, and corresponding reference for each entry is provided in Supplementary Table S1.

**Table 2:** Antioxidant activities of *C. longa* extracts, essential oils, and isolated curcuminoids evaluated using different in vitro analytical models.

Analytical Models	*C. longa* Materials	Key Results	References
DPPH radical scavenging	Rhizome essential oil	51.45 ± 0.59% scavenging at 150 µg/mL (mean ± SD; *n* = 3)	[86]
	Fresh-rhizome essential oil	IC_50_ = 10.03 mg/mL	[87]
	Ethanolic rhizome extract, Khulna “mura” variety	IC_50_ = 1.08 µg/mL (assays performed in triplicate); stronger activity was generally observed in ethanolic than aqueous extracts	[85]
	Methanolic extract of the Ryudai Gold variety	IC_50_ = 26.4 µg/mL	[90]
	Leaf essential oil	IC_50_ = 8.62 ± 0.18 µg/mL (mean ± SD; *n* = 6, performed in triplicate)	[39]
ABTS radical scavenging	Fresh-rhizome essential oil	IC_50_ = 0.54 mg/mL	[87]
	Leaf essential oil	IC_50_ = 9.21 ± 0.29 µg/mL (mean ± SD; *n* = 6, performed in triplicate)	[39]
	Isolated rhizome curcuminoids and formulated complexes	Activity order: quercetin > Trolox > curcuminoids > curcumin–cyclodextrin complex > curcumin–phospholipid complex	[83]
FRAP/ferric-reducing activity	Rhizome essential oil	Concentration-dependent reducing power; activity lower than BHA and BHT but greater than ginger oil	[86]
	Curcuminoids and formulated complexes	Reducing-power order: quercetin > Trolox > curcumin–cyclodextrin complex > curcuminoids > curcumin–phospholipid complex	[83]
	Ethanolic extract, Khulna “chora” variety	4204.46 ± 74.48 µM Fe(II)/100 g (mean ± SD; *n* = 3; *p* < 0.05 for between-extract comparisons)	[85]
CUPRAC	Rhizome essential oil	3.40 ± 0.071 mmol Trolox equivalents/g oil (mean ± SD; *n* = 3)	[86]
ORAC	Methanolic extract of the Ryudai Gold variety	14,090 µmol Trolox equivalents/g extract	[90]
Hydroxyl-radical/2-deoxyribose assay	Methanolic extract of the Ryudai Gold variety	IC_50_ = 7.4 µg/mL	[90]
Hydrogen peroxide scavenging	Leaf essential oil	IC_50_ = 4.35 ± 0.16 µg/mL (mean ± SD; *n* = 6, performed in triplicate)	[39]
HOSC, RDSC, and ABTS capacity	Ethanolic rhizome extract	HOSC 1524.59, RDSC 56.38, and ABTS 1.70 µmol Trolox equivalents/g; TPC 27.12 mg GAE/g	[91]
Phosphomolybdenum and linoleic acid peroxidation	Isolated individual curcuminoids	Antioxidant potency generally followed curcumin > demethoxycurcumin > bisdemethoxycurcumin	[84]

DPPH radical scavenging: 2,2-Diphenyl-1-picrylhydrazyl radical-scavenging assay; ABTS radical scavenging: 2,2′-Azino-bis(3-ethylbenzothiazoline-6-sulfonic acid) radical-cation-scavenging assay; FRAP/ferric-reducing activity: Ferric Reducing Antioxidant Power assay; CUPRAC: Cupric Ion Reducing Antioxidant Capacity assay; ORAC: Oxygen Radical Absorbance Capacity assay; Hydroxyl-radical/2-deoxyribose assay: Hydroxyl Radical-Scavenging Activity Assessed by the 2-Deoxyribose Oxidation Assay; HOSC: Hydroxyl Radical-Scavenging Capacity; RDSC: DPPH Radical-Scavenging Capacity; ABTS capacity: ABTS Radical-Cation-Scavenging Capacity.

**Table 3:** Antimicrobial activities of *C. longa*, its extracts, essential oils, curcuminoids, and formulations.

Microorganism Type	Microorganisms	Tested Materials	Key Results	References
Filamentous fungus	*Aspergillus flavus*	Rhizome n-hexane extract	The extract showed detectable antifungal activity	[61]
	*Aspergillus fumigatus*	Leaf essential oil	Inhibition zone approximately 25 mm at 500 µg/mL	[129]
	*Aspergillus niger*	n-hexane rhizome extract	The extract showed detectable antifungal activity	[61]
Gram-negative anaerobic bacterium	*Fusobacterium nucleatum* ATCC 23726	Purified curcumin	MIC 10 µg/mL	[130]
	*Porphyromonas gingivalis* ATCC 33277	Purified curcumin	MIC 15 µg/mL	[130]
		Curcumin containing ≥95% curcuminoids	MIC 62.5 µg/mL MBC 125 µg/mL	[122]
		Curcumin	Dose-dependent inhibition of bacterial adhesion and biofilm formation	[122]
	*Porphyromonas gingivalis* OMZ314	Purified curcumin	MIC 10 µg/mL; >80% inhibition of homotypic and mixed biofilms at 20 µg/mL	[130]
	*Prevotella intermedia* ATCC 49046	Purified curcumin	MIC 10 µg/mL	[130]
Gram-negative bacterium	*Acinetobacter lwoffii*	Purified curcumin	MIC approximately 250 µg/mL	[121]
	*Aeromonas hydrophila*	Curcuminoids	MIC 250 ppt	[131]
	*Aggregatibacter actinomycetemcomitans* ATCC 29522 and ATCC 29523	Purified curcumin	MIC > 100 µg/mL	[130]
	*Citrobacter gillenii*	Rhizome n-hexane extract	Inhibition zone 28 ± 1.03 mm	[120]
	*Edwardsiella tarda*	Curcuminoids	MIC 500 ppt	[131]
	*Escherichia coli*	Bulk curcumin and nanocurcumin	Inhibition zones of 19.70 ± 1.18 and 24.58 ± 1.12 mm, respectively	[124]
		Combined turmeric and ginger rhizome volatile oils at a 1:1 ratio	Inhibition zone 22.00 ± 1.26 mm, greater than that produced by either oil alone	[132]
		Leaf essential oil	Inhibition zone approximately 22.2 mm	[39]
		Rhizome volatile oil	Inhibition zone 19.00 ± 1.26 mm at 50 µL	[132]
	*Escherichia coli* ATCC 11303	Rhizome extracts	Organic extracts showed antibacterial activity at 5 mg/mL; the aqueous extract was inactive	[61]
	*Escherichia coli* ATCC 25922	Purified curcumin	MIC 163 µg/mL	[133]
	*Klebsiella ornithinolytica*	Rhizome n-hexane extract	Inhibition zone 26 ± 1.63 mm	[120]
		Rhizome volatile oil	MIC was 6.25 mg/mL	[120]
	*Klebsiella pneumoniae*	Rhizome ethanol extract	Growth was inhibited at 10 mg turmeric equivalents/mL	[91]
		Rhizome volatile oil	Inhibition zone 17.00 ± 1.11 mm at 50 µL	[132]
	*Klebsiella pneumoniae* ATCC 13883	Purified curcumin	MIC 62.5 µg/mL MBC 125 µg/mL	[134]
	*Klebsiella pneumoniae* ATCC 700603	Purified curcumin	MIC 216 µg/mL	[133]
		Ethanolic and methanolic dried-rhizome extracts	MIC 32 µg/mL for both extracts	[135]
	*Proteus vulgaris* ATCC 3851	Purified curcumin	MIC 62.5 µg/mL MBC 62.5 µg/mL	[134]
	*Pseudomonas aeruginosa*	Leaf essential oil	Inhibition zone approximately 20.4 mm	[39]
		Purified curcumin	MICs 62.5 µg/mL	[121]
	*Pseudomonas aeruginosa* ATCC 27853	Purified curcumin	MIC 175 µg/mL	[133]
		Purified curcumin	MIC 62.5 µg/mL MBC 125 µg/mL	[134]
	*Salmonella enterica*	Leaf essential oil	Inhibition zone approximately 17.6 mm	[39]
Gram-negative biofilm-forming bacterium	*Pseudomonas aeruginosa*, clinical isolates	Ethanolic rhizome extract	Biofilm inhibition 26.76–58.55% at 0.5–2 mg/mL	[136]
	*Pseudomonas aeruginosa* MTCC 3541	Lakadong-derived curcumin-loaded PLGA–carbopol nanogel	Disrupted established biofilm at 1000 µg/mL	[125]
Gram-negative multidrug-resistant bacterium	*Escherichia coli*, MDR isolates	Purified curcumin	MICs ≥ 2000 µg/mL	[121]
	*Proteus mirabilis*, MDR isolates	Purified curcumin	MICs ≥ 2000 µg/mL	[121]
Gram-positive bacterium	*Bacillus subtilis*	Rhizome-derived curcuminoids	MIC 125 ppt	[131]
	*Bacillus subtilis ATCC 6633*	Purified curcumin	MIC 129 µg/mL	[133]
	*Enterococcus faecalis*	Purified curcumin	MICs 62.5 µg/mL	[121]
	*Enterococcus faecalis* ATCC 11700	Turmeric extracts	MIC 512–1024 µg/mL	[137]
		Purified curcumin	MIC 1024 µg/mL	[137]
	*Enterococcus faecalis* ATCC 29212	Purified curcumin	MIC 293 µg/mL	[133]
		Purified curcumin	MIC 7.81 µg/mL MBC 31.25 µg/mL	[134]
	*Listeria monocytogenes* ATCC 7644	Purified curcumin	MIC 62.5 µg/mL MBC 62.5 µg/mL	[134]
	*Staphylococcus aureus*	Bulk curcumin	Inhibition zones of 24.82 ± 0.54 mm	[124]
		nanocurcumin	Inhibition zones of 29.91 ± 0.53 mm	[124]
		Leaf essential oil	Inhibition zone 20.6 mm	[39]
		Leaf essential oil	Inhibition zone 18 mm at 500 µg/mL	[129]
		Rhizome n-hexane extract	Inhibition zone 25 ± 0.33 mm	[120]
		Rhizome ethanol extract	Growth was inhibited at 0.1 and 1 mg turmeric equivalents/mL	[91]
		Rhizome volatile oil	Inhibition zone 19.00 ± 1.23 mm at 50 µL	[132]
	*Staphylococcus aureus* ATCC 25923	Curcuminoid fraction containing 86.5% curcumin	MIC 3.91 ppt	[131]
		Dried rhizome ethyl acetate, methanol, and aqueous extracts	MICs 2, 8, and 64 mg/mL, respectively	[138]
		Purified curcumin	MIC 62.5 µg/mL MBC 62.5 µg/mL	[134]
		Turmeric extracts and Purified curcumin	Extract MIC 128–256 µg/mL; curcumin MIC 128 µg/mL	[137]
	*Staphylococcus epidermidis*	Rhizome-derived curcuminoids	MIC 125 ppt	[131]
	*Staphylococcus intermedius*	Rhizome-derived curcuminoids	MIC 125 ppt	[131]
	*Streptococcus agalactiae*	Rhizome-derived curcuminoids	MIC 500 ppt	[131]
	*Streptococcus mutans*	Leaf essential oil	Inhibition zone approximately 18 mm at 500 µg/mL	[129]
	*Streptococcus pyogenes*	Purified curcumin	MIC 31.25 µg/mL	[121]
	*Staphylococcus aureus*, clinical isolates	Rhizome ethanol extract	MIC 10 mg/mL; biofilm inhibition 48.86–77.72% at 0.5–2 mg/mL	[136]
	*Staphylococcus aureus*, wound-derived isolate	Purified curcumin	Biofilm eradication 94.56 ± 5.60% at 150 µg/mL	[139]
Gram-positive methicillin-resistant bacterium	MRSA clinical isolate	Rhizome ethanol extract	MIC 125 µg/mL	[140]
	MRSA DPS-1	Bisdemethoxycurcumin combined with gentamicin	Gentamicin MIC reduced 16-fold; FICI 0.10; complete growth inhibition after 8 h at MIC of each agent	[127]
Gram-positive multidrug-resistant bacterium	*Staphylococcus aureus*, MDR isolates	Purified curcumin	MICs ≥ 2000 µg/mL	[121]
	*Staphylococcus haemolyticus*, MDR isolates	Purified curcumin	MICs ≥ 2000 µg/mL	[121]
Gram-positive spore-forming bacterium	*Bacillus cereus*	Rhizome-derived curcuminoids	MIC 15.63 ppt	[131]
	*Bacillus cereus* ATCC 11778	Purified curcumin	MIC 62.5 µg/mL MBC 125 µg/mL	[134]
Spirochete	*Treponema denticola* ATCC 33520	Purified curcumin	MIC 5 µg/mL	[130]
Yeast	*Candida albicans* ATCC 10231	Purified curcumin	MIC 512 µg/mL	[137]

EPS, extracellular polymeric substances; FICI, fractional inhibitory concentration index; MBC, minimum bactericidal concentration; MBIC, minimum biofilm inhibitory concentration; MDR, multidrug-resistant; MIC, minimum inhibitory concentration; MRSA, methicillin-resistant Staphylococcus aureus; MSSA, methicillin-susceptible *S. aureus*; PBP2a, penicillin-binding protein 2a.

**Table 4:** Antiviral activities of *C. longa*-derived materials, curcumin, and curcumin analogues against RNA viruses.

Virus	Tested *C. longa* Materials	Key Antiviral Results	References
Dengue virus serotype 2 (DENV-2)	Synthetic curcumin analogue 6	- EC_50_ = 12.5 µM; SI = 1.42 (mean ± SD).	[159]
	Synthetic curcumin analogue 7	- EC_50_ = 15 µM; SI = 1.20 (mean ± SD).	[159]
	Curcumin	- EC_50_ = 13.63 ± 0.95 µM (mean ± SD); SI = 1.67 in Vero cells	[159]
Hepatitis C virus, genotypes 1–7	Curcumin derived from rhizome	- Curcumin inhibited entry of all major HCV genotypes with IC_50_ = 8.46 ± 1.27 µM, equivalent to 2.94 ± 0.37 µg/mL.	[146]
Human immunodeficiency virus type 1 (HIV-1)	Synthetic curcumin analogue “curcumin A”	Curcumin A and curcumin each produced IC_50_ = 0.7 µM in CEM-T cells.	[151]
Influenza A virus A/Puerto Rico/8/34 (H1N1)	Curcumin	- Network pharmacology identified AKT1, RELA, MAPK1, and TP53 as key candidate targets. - Curcumin showed the strongest predicted interaction with AKT1 and reduced influenza-induced AKT expression in MDCK cells (Three independent experiments for in vitro validation; statistical significance reported where applicable).	[155]
	Curcumin-loaded self-emulsion micelle formulation, Cur-M	- Cur-M inhibited infection after viral entry, suppressed influenza M-segment RNA and minigenome replication, and reduced Akt phosphorylation (experiments performed in triplicate; viral RNA data from three independent experiments; *p* < 0.05 where indicated).	[160]
	Curcumin and monoacetylcurcumin	- Both compounds inhibited viral production. Monoacetylcurcumin suppressed Akt phosphorylation.	[156]
	Curcumin, tetrahydrocurcumin, and petasiphenol	- Direct plaque-reduction EC_50_ values were 0.17 µM for curcumin and 14.65 µM for petasiphenol. - The conjugated double bonds in curcumin were important for direct antiviral activity.	[161]
	Water-solubilized curcuminoid–stevioside nanoparticles, C–S/M	- C–S/M suppressed viral NS1 RNA at lower curcuminoid-equivalent concentrations than native curcuminoids and reduced TNF-α, IL-6, and IL-8. - Oral treatment at 100–400 mg/kg/day reduced pulmonary viral burden and restored Th1, activated CD8^+^, and multifunctional T-cell responses in infected mice.	[162]
Influenza A virus H1N1 neuraminidase	Thirteen curcuminoids isolated from methanolic rhizome extract	- All isolated compounds acted as noncompetitive neuraminidase inhibitors, with IC_50_ values ranging from 6.18 ± 0.64 to 40.17 ± 0.79 µg/mL.	[150]
Influenza A virus, novel H1N1 wild-type neuraminidase	Demethoxycurcumin, bisdemethoxycurcumin, and curcumin isolated from rhizome	- IC_50_ values were 4.36 ± 0.57, 6.95 ± 0.92, and 3.46 ± 0.27 µg/mL for demethoxycurcumin, bisdemethoxycurcumin, and curcumin, respectively.	[150]
Influenza A virus, oseltamivir-resistant H1N1 H274Y neuraminidase	Demethoxycurcumin, bisde-methoxycurcumin, and curcu-min isolated from rhizome	- The three curcuminoids retained inhibitory activity against H274Y neuraminidase, showing only approximately 1.88- to 2.59-fold decreases in potency.	[150]
Japanese encephalitis virus (JEV)	Curcumin	- Pre-incubation with curcumin reduced plaque formation by disrupting the viral lipid envelope (*n* = 3 independent experiments) - direct treatment at 30 µM markedly reduced infectivity.	[143]
SARS-CoV-2	Aqueous rhizome extract	- A 1:128 dilution significantly neutralized SARS-CoV-2 in human Calu-3 cells. - Complete neutralization was also observed in Vero E6 cells at lower dilutions.	[149]
	Curcumin-containing turmeric supplement material	- A concentration of 58.6 µg/mL significantly reduced infection in Calu-3 cells.	[149]
	Pure curcumin	- Curcumin at 15.6 µg/mL significantly neutralized SARS-CoV-2 in Calu-3 cells. - RT-qPCR analysis of Vero E6 supernatants showed an EC_50_ of approximately 14 µg/mL for reduction in viral RNA.	[149]
Yellow fever virus (YFV-17DD)	Synthetic curcumin analogue 6	- EC_50_ = 10.85 ± 0.13 µM (mean ± SD); SI = 1.63.	[159]
	Synthetic curcumin analogue 7	- EC_50_ = 11.94 ± 0.39 µM (mean ± SD); SI = 1.51.	[159]
Zika virus (ZIKV)	Curcumin and synthetic curcumin analogues	- Curcumin and analogues 2, 6, and 7 showed activity, with EC_50_ values ranging from 4.04 ± 0.38 to 32.45 ± 1.58 µM - SI values ranging from 4.45 to 1.33. Analogues 6 and 7 were active against ZIKV, DENV-2, and YFV.	[159]

AKT1, AKT serine/threonine kinase 1; C–S/M, water-solubilized curcuminoid–stevioside nanoparticle formulation prepared using microwave-assisted treatment; CD8^+^, cluster of differentiation 8-positive T cell; CEM-T, human T-lymphoblastoid cell line; Cur-M, curcumin-loaded self-emulsion micelle formulation; DENV-2, dengue virus serotype 2; EC_50_, half-maximal effective concentration; H1N1, influenza A virus subtype containing hemagglutinin type 1 and neuraminidase type 1; H274Y, histidine-to-tyrosine substitution at residue 274 of influenza neuraminidase; HCV, hepatitis C virus; HIV-1, human immunodeficiency virus type 1; IC_50_, half-maximal inhibitory concentration; IL, interleukin; JEV, Japanese encephalitis virus; MAPK1, mitogen-activated protein kinase 1; MDCK, Madin–Darby canine kidney; NS1, nonstructural protein 1; RELA, RELA proto-oncogene, NF-κB subunit; RT-qPCR, reverse transcription quantitative polymerase chain reaction; SARS-CoV-2, severe acute respiratory syndrome coronavirus 2; SI, selectivity index; Th1, T helper type 1 cell; TNF-α, tumor necrosis factor alpha; TP53, tumor protein p53; YFV-17DD, yellow fever virus 17DD vaccine strain; ZIKV, Zika virus.

## Data Availability

No new data were created or analyzed in this study. Data sharing is not applicable to this article.

## Supplementary Materials

The following supporting information can be downloaded at https://www.mdpi.com/article/10.3390/ph19091480/s1, Table S1: Analytical identification and structural confirmation of phytochemical constituents reported from different parts of *Curcuma longa*.

## Abbreviations

The following abbreviations are used in this manuscript:

ABTS	2,2′-Azino-bis(3-ethylbenzothiazoline-6-sulfonic acid)
ACC	Acetyl-CoA carboxylase
AKT/Akt	Protein kinase B
AKT1	AKT serine/threonine kinase 1
ALT	Alanine aminotransferase
AMPK	AMP-activated protein kinase
AP-1	Activator protein 1
AST	Aspartate aminotransferase
Bcl-2	B-cell lymphoma 2
BDMC	Bisdemethoxycurcumin
BHA	Butylated hydroxyanisole
BHT	Butylated hydroxytoluene
CAS	Chemical Abstracts Service
CCL7	C-C motif chemokine ligand 7
CCR2	C-C chemokine receptor type 2
CD	Cluster of differentiation
cFLIP	Cellular FLICE-like inhibitory protein
cGAS	Cyclic GMP–AMP synthase
CK	Creatine kinase
CMPK2	Cytidine/uridine monophosphate kinase 2
COX	Cyclooxygenase
CPT-1	Carnitine palmitoyltransferase 1
CRP	C-reactive protein
CRYAB	Alpha B-crystallin
CUPRAC	Cupric Ion Reducing Antioxidant Capacity
CXCR4	C-X-C chemokine receptor type 4
DENV-2	Dengue virus serotype 2
DESI-MSI	Desorption electrospray ionization mass spectrometry imaging
DPP-4	Dipeptidyl peptidase-4
DPPH	2,2-Diphenyl-1-picrylhydrazyl
DR4	Death receptor 4
DR5	Death receptor 5
EC_50_	Half-maximal effective concentration
EGFR	Epidermal growth factor receptor
EMT	Epithelial–mesenchymal transition
ENPP2	Ectonucleotide pyrophosphatase/phosphodiesterase 2
EPS	Extracellular polymeric substances
ER	Endoplasmic reticulum
ERK	Extracellular signal-regulated kinase
FAK	Focal adhesion kinase
FAS	Fatty acid synthase
FATP	Fatty acid transport protein
FICI	Fractional inhibitory concentration index
FRAP	Ferric Reducing Antioxidant Power
GAE	Gallic acid equivalents
GATA3	GATA-binding protein 3
GBF1	Golgi brefeldin A-resistant guanine nucleotide exchange factor 1
GLUT2	Glucose transporter 2
GLUT4	Glucose transporter 4
GSK3β	Glycogen synthase kinase 3 beta
HBV	Hepatitis B virus
HBsAg	Hepatitis B surface antigen
HbA1c	Glycated hemoglobin
HCV	Hepatitis C virus
HIF-1α	Hypoxia-inducible factor 1 alpha
HIV-1	Human immunodeficiency virus type 1
HO-1	Heme oxygenase-1
HOMA-IR	Homeostatic Model Assessment for Insulin Resistance
HOSC	Hydroxyl Radical-Scavenging Capacity
hs-CRP	High-sensitivity C-reactive protein
IC_50_	Half-maximal inhibitory concentration
ICAM-1	Intercellular adhesion molecule 1
IgE	Immunoglobulin E
IL	Interleukin
IP-10	Interferon gamma-induced protein 10
JAK	Janus kinase
JEV	Japanese encephalitis virus
JNK	c-Jun N-terminal kinase
KOOS	Knee injury and Osteoarthritis Outcome Score
LDH-A	Lactate dehydrogenase A
MAPK	Mitogen-activated protein kinase
MBC	Minimum bactericidal concentration
MBIC	Minimum biofilm inhibitory concentration
MCP-1	Monocyte chemoattractant protein 1
MDR	Multidrug-resistant
MHC	Major histocompatibility complex
MIC	Minimum inhibitory concentration
MIP-1α	Macrophage inflammatory protein 1 alpha
MMP	Matrix metalloproteinase
MRSA	Methicillin-resistant Staphylococcus aureus
MSSA	Methicillin-susceptible Staphylococcus aureus
mTOR	Mechanistic target of rapamycin
MyD88	Myeloid differentiation primary response 88
NAFLD	Non-alcoholic fatty liver disease
NASH	Non-alcoholic steatohepatitis
NF-κB	Nuclear factor kappa B
NLRP3	NOD-like receptor family pyrin domain-containing 3
NOD2	Nucleotide-binding oligomerization domain-containing protein 2
NPC1L1	Niemann–Pick C1-like 1
Nrf2	Nuclear factor erythroid 2-related factor 2
NS1	Nonstructural protein 1
NTCP	Sodium taurocholate cotransporting polypeptide
ORAC	Oxygen Radical Absorbance Capacity
PAK1	p21-activated kinase 1
PBP2a	Penicillin-binding protein 2a
PDE-4B	Phosphodiesterase 4B
PDGFRβ	Platelet-derived growth factor receptor beta
PGE_2_	Prostaglandin E_2_
PI3K	Phosphoinositide 3-kinase
PI4KB	Phosphatidylinositol 4-kinase beta
PKCα	Protein kinase C alpha
PLGA	Poly(lactic-co-glycolic acid)
PPAR-α	Peroxisome proliferator-activated receptor alpha
PPAR-γ	Peroxisome proliferator-activated receptor gamma
PTP1B	Protein tyrosine phosphatase 1B
RDSC	DPPH Radical-Scavenging Capacity
RIG-I	Retinoic acid-inducible gene I
RORγt	Retinoic acid receptor-related orphan receptor gamma t
ROS	Reactive oxygen species
RT-qPCR	Reverse transcription quantitative polymerase chain reaction
SARS-CoV-2	Severe acute respiratory syndrome coronavirus 2
SDG	Sustainable Development Goal
SI	Selectivity index
SIRT1	Sirtuin 1
SOD	Superoxide dismutase
SREBP	Sterol regulatory element-binding protein
STAT	Signal transducer and activator of transcription
α-SMA	Alpha-smooth muscle actin
TCM	Traditional Chinese Medicine
Th	T helper
TIMP-1	Tissue inhibitor of metalloproteinases 1
TLR	Toll-like receptor
TNF-α	Tumor necrosis factor alpha
TPC	Total phenolic content
TP53	Tumor protein p53
UPLC-MS	Ultra-performance liquid chromatography–mass spectrometry
UHPLC-Q-Orbitrap HRMS	Ultra-high-performance liquid chromatography–Q-Orbitrap high-resolution mass spectrometry
VAS	Visual Analog Scale
VCAM-1	Vascular cell adhesion molecule 1
VEGF	Vascular endothelial growth factor
VEGFR2	Vascular endothelial growth factor receptor 2
WOMAC	Western Ontario and McMaster Universities Osteoarthritis Index
XIAP	X-linked inhibitor of apoptosis protein
YFV	Yellow fever virus
ZIKV	Zika virus

## Appendix A

The different plant parts of *C. longa* and their botanical definitions are summarized in Table A1.

**Table A1:** Botanical definitions of major organs of *C. longa*.

Plant Organ	Definition
Rhizome	A modified underground stem characterized by nodes, internodes, scale leaves, buds, and adventitious roots. It serves mainly as a storage and vegetative propagation organ.
Root	A true underground organ arising from the rhizome and lacking nodes, internodes, scale leaves, and buds. It functions mainly in anchorage and the uptake of water and minerals.
Root tuber	A swollen storage structure formed by enlargement of a true adventitious root, usually toward its distal portion, and lacking stem nodes and buds.
Leaf	An aerial photosynthetic organ composed of a leaf sheath, petiole, and lamina. The overlapping basal leaf sheaths contribute to formation of the pseudostem.
Pseudostem	A stem-like aerial structure formed by tightly overlapping leaf sheaths rather than by a true anatomical stem.
Inflorescence/flower	The reproductive structure consisting of a spike with overlapping bracts that enclose individual flowers arising from the underground rhizome.

The phytochemical composition of *C. longa* varies among plant organs. The rhizome is the most extensively characterized and contains diverse curcuminoids, phenolic compounds, terpenoids, fatty acids, sterols, and other constituents. Leaf and flower are mainly characterized by volatile mono- and sesquiterpenoids, whereas roots and root tuber remains less studied but contain distinct phenolic derivatives, alkaloids, fatty acids, and volatile terpenoids. These organ-specific differences highlight the importance of distinguishing rhizome, root, and root tuber when discussing the phytochemistry of *C. longa*.
