# Supplementary material for: Beyond the Rhizome: Phytochemistry, Biological Activities, and Sustainable Utilization of Curcuma longa L
Source: Pharmaceuticals (Basel). 2026 Sep 17;19(9):1480. doi: 10.3390/ph19091480 (PMC13610708; doi:10.3390/ph19091480)
Supplement: Supplementary file 1 [file pharmaceuticals-19-01480-s001.zip › pharmaceuticals-4549339-supplementary.pdf]

Beyond the Rhizome: Phytochemistry, Biological Activities, and Sustainable Utilization of *Curcuma longa* L.

Kannika Thongkhao, Siripat Chaichit, Santhosh Kumar J Urumarudappa and Aekkhaluck Intharuksa*

Supplementary materials

Table S1. Analytical identification and structural confirmation of phytochemical constituents reported from different parts of *Curcuma longa*

Phytochemical group	Compound	Plant part	Identification method / analytical basis	Level of structural confirmation	Reference(s)
Curcuminoids and diarylheptanoids	1,5-bis(4-hydroxy-3-methoxyphenyl)-penta-(1E,4E)-1,4-dien-3-one	root tuber	Isolation and chromatographic purification; spectroscopic analysis (NMR/MS)	Isolated; structure elucidated by spectroscopy	[40]
	1,5-dihydroxy-1,7-bis(4-hydroxy-3-methoxyphenyl)-4,6-heptadiene-3-one	rhizome	Isolation; 1D/2D NMR spectroscopy	Structure elucidated and assigned by NMR	[41]
	1,5-dihydroxy-1,7-bis(4-hydroxyphenyl)-4,6-heptadiene-3-one	rhizome	Isolation; 1D/2D NMR spectroscopy	Structure elucidated and assigned by NMR	[41]
	1,5-dihydroxy-1-(4-hydroxy-3-methoxyphenyl)-7-(4-hydroxyphenyl)-4,6-heptadiene-3-one	rhizome	Isolation; 1D/2D NMR spectroscopy	Structure elucidated and assigned by NMR	[41]
	1,5-dihydroxy-1-(4-hydroxyphenyl)-7-(4-hydroxy-3-methoxyphenyl)-4,6-heptadiene-3-one	rhizome	Isolation; 1D/2D NMR spectroscopy	Structure elucidated and assigned by NMR	[41]
	1,7-bis(4-hydroxyphenyl)-1-heptene-3,5-dione	rhizome	Isolation; 1D/2D NMR spectroscopy	Structure elucidated and assigned by NMR	[41]
	1-(4-hydroxy-3-methoxyphenyl)-7-(3,4-dihydroxyphenyl)-1,6-heptadiene-3, 5-dione	rhizome	Isolation and chromatographic purification; spectroscopic analysis	Isolated and structurally identified	[42]
	1-(4-hydroxy-3-methoxyphenyl)-5-(4-hydroxyphenyl)-1,4-pentadiene-3-one	rhizome	Isolation; 1D/2D NMR spectroscopy	Structure elucidated and assigned by NMR	[41]
	3-hydroxy-1,7-bis-(4-hydroxyphenyl)-6-heptene-1,5-dione	rhizome	Isolation; 1D/2D NMR spectroscopy	Structure elucidated and assigned by NMR	[41]
	5-hydroxyl-1-(4-hydroxy-3-methoxyphenyl)-7-(4-hydroxyphenyl)-4,6-heptadiene-3-one	rhizome	Isolation; 1D/2D NMR spectroscopy	Structure elucidated and assigned by NMR	[41]
	5-hydroxyl-1,7-bis(4-hydroxy-3-methoxyphenyl)-4,6-heptadiene-3-one	rhizome	Isolation and chromatographic purification; spectroscopic analysis	Isolated and structurally identified	[42]
	5-hydroxyl-7-(4-hydroxy-3-methoxyphenyl)-1-(4-hydroxyphenyl)-4,6-heptadiene-3-one	rhizome	Isolation and chromatographic purification; spectroscopic analysis	Isolated and structurally identified	[42]
	bisdemethoxycurcumin	rhizome	HPTLC with densitometric analysis/reference standards	Chromatographic identification/quantification by comparison with standards	[43]
			Secondary review; not reported	Secondary review; not reported	[44]
			HPLC-ESI-MS/MS and GNPS molecular networking	Putative annotation based on MS/MS fragmentation and molecular networking	[45]
	curcumin	rhizome	Secondary review; not reported.	Secondary review; not reported	[44]
			HPLC–ESI–MS/MS; MS/MS fragmentation with molecular networking	Putative annotation based on MS/MS fragmentation and molecular networking	[45]
			HPLC-MS/MS with high-resolution MS/MS and molecular networking (GNPS) for metabolite annotation.	Putative/tentative metabolite annotation.	[46]
		not reported	Secondary review; not reported	Secondary review; not reported.	[47]

Phytochemical group	Compound	Plant part	Identification method / analytical basis	Level of structural confirmation	Reference(s)
	cyclocurcumin	not reported	HPLC quantification of *C. longa* extract	Detected/quantified chromatographically; structural confirmation not reported	[48]
		rhizome	HPLC-UV-Vis fingerprinting	Chromatographic peak assignment; structural confirmation not reported	[49]
	demethoxycurcumin	rhizome	Secondary review; not reported.	Secondary review; not reported.	[44]
			HPLC-ESI-MS/MS and GNPS molecular networking	Putative annotation based on MS/MS fragmentation and molecular networking	[45]
			Secondary review; not reported.	Secondary review; not reported.	[46]
	methylcurcumin	rhizome	Secondary review; not reported.	Secondary review; not reported.	[46]
	monodemethylcurcumin	rhizome	HPLC-ESI-MS/MS and GNPS molecular networking	Putative annotation based on MS/MS fragmentation and molecular networking	[45]
	octahydrocurcumin	leaf	Bioassay-guided isolation; preparative HPLC; 1H/13C NMR	Isolated; structure confirmed by NMR	[50]
	sodium curcuminate	rhizome	Secondary review; not reported.	Secondary review; not reported.	[46]
	tetrahydroxycurcumin	rhizome	Isolation; spectroscopic analysis	Isolated; characterized by spectroscopic analysis	[51]
Other phenolic compounds and phenylpropanoid derivatives	(E)-ferulic acid	rhizome	Isolation and chromatographic purification; spectroscopic analysis	Isolated; structure elucidated by spectroscopy	[52]
	(Z)-ferulic acid	rhizome	Isolation and chromatographic purification; spectroscopic analysis	Isolated; structure elucidated by spectroscopy	[52]
	2-methoxy-4-vinylphenol	rhizome	GC-MS for volatile constituents; column chromatography with IR and 1H/13C NMR for isolated curcumin	GC-MS-based identification for volatile constituents; isolated curcumin confirmed by IR and NMR	[48]
	4-hydroxycinnamic acid	rhizome	HPLC-ESI-MS/MS and GNPS molecular networking	Putative annotation based on MS/MS fragmentation and molecular networking	[45]
	calebin A	rhizome	Secondary review; not reported.	Secondary review; not reported.	[44]
	dehydrozingerone	root, rhizome	HPLC-UV-Vis fingerprinting	Chromatographic peak assignment; structural confirmation not reported	[49]
			Isolation and chromatographic purification; spectroscopic analysis	Isolated; structure elucidated by spectroscopy	[52]
	eugenol	leaf, rhizome	GC-MS for volatile constituents; column chromatography with IR and 1H/13C NMR for isolated curcumin	GC-MS-based identification for volatile constituents; isolated curcumin confirmed by IR and NMR	[53]

Phytochemical group	Compound	Plant part	Identification method / analytical basis	Level of structural confirmation	Reference(s)
			GC analysis of leaf essential oils	GC-based identification; detailed structural confirmation not reported	[54]
			GC-MS and GC-FID	GC-MS/retention-data based identification	[55]
	vanillic acid	rhizome	Isolation and chromatographic purification; spectroscopic analysis	Isolated; structure elucidated by spectroscopy	[52]
	vanillin	rhizome	HPLC-ESI-MS/MS and GNPS molecular networking	Putative annotation based on MS/MS fragmentation and molecular networking	[45]
			Isolation and chromatographic purification; spectroscopic analysis	Isolated; structure elucidated by spectroscopy	[52]
	zingerone	root tuber	Isolation from root tuber	Isolated; detailed structural confirmation not reported.	[56]
Monoterpenoids	(−)-terpinen-4-ol	rhizome	GC-MS fingerprinting with chemometric analysis	GC-MS identification based on mass-spectral/retention information	[57]
	(E)-β-ocimene	rhizome	Secondary review; not reported.	Secondary review; not reported.	[58]
	(Z)-β-ocimene (cis-ocimene)	rhizome	Gas chromatography (GC)	GC-based identification; detailed structural confirmation not reported	[59]
		leaf, rhizome	GC/GC-MS analysis of leaf and rhizome essential oils	GC/GC-MS-based identification; detailed structural confirmation not reported	[60]
	1,8-cineol (eucalyptol)	rhizome	GC-MS for volatile constituents; column chromatography with IR and 1H/13C NMR for isolated curcumin	GC-MS-based identification for volatile constituents; isolated curcumin confirmed by IR and NMR	[53]
		rhizome	GC-MS and GC-FID	GC-MS/retention-data based identification	[55]
		rhizome	Secondary review; not reported.	Secondary review; not reported.	[58]
		leaf, rhizome	GC/GC-MS analysis of leaf and rhizome essential oils	GC/GC-MS-based identification; detailed structural confirmation not reported	[60]
	2-isopropylidene-3-methylhexa-3,5-dienal	rhizome	GC-MS for volatile constituents; column chromatography with IR and 1H/13C NMR for isolated curcumin	GC-MS-based identification for volatile constituents; isolated curcumin confirmed by IR and NMR	[53]
	2-menthen-1-ol	leaf	Bioassay-guided isolation; preparative HPLC; 1H/13C NMR	Isolated; structure confirmed by NMR	[50]
	3,7,7-trimethyl-1,3,5-cycloheptatriene	rhizome	HPLC analysis of curcuminoids; GC-MS analysis of volatile compounds	Chromatographic/MS-based identification; detailed structural confirmation not reported	[61]

Phytochemical group	Compound	Plant part	Identification method / analytical basis	Level of structural confirmation	Reference(s)
	3-carene	rhizome, root tuber	GC-MS fingerprinting with chemometric analysis	GC-MS identification based on mass-spectral/retention information.	[57]
	4-isopropenyl-1,2-dimethylcyclohex-ane-2-enol	rhizome	GC-MS for volatile constituents; column chromatography with IR and 1H/13C NMR for isolated curcumin	GC-MS-based identification for volatile constituents; isolated curcumin confirmed by IR and NMR	[53]
	α-fenchene	flower, leaf	Not reported.	Not reported.	[9]
	α-phellantrene	rhizome, root tuber	GC-MS fingerprinting with chemometric analysis	GC-MS identification based on mass-spectral/retention information	[57]
		leaf, rhizome	GC/GC-MS analysis of leaf and rhizome essential oils	GC/GC-MS-based identification; detailed structural confirmation not reported	[60]
	α-pinene	leaf	GC analysis of leaf essential oils	GC-based identification; detailed structural confirmation not reported	[54]
		rhizome	Gas chromatography (GC)	GC-based identification; detailed structural confirmation not reported	[59]
	α-terpinene	rhizome, root tuber	GC-MS fingerprinting with chemometric analysis	GC-MS identification based on mass-spectral/retention information	[57]
	α-terpineol	rhizome, root tuber	GC-MS fingerprinting with chemometric analysis	GC-MS identification based on mass-spectral/retention information	[57]
	α-thujone	rhizome	GC-MS for volatile constituents; column chromatography with IR and 1H/13C NMR for isolated curcumin	GC-MS-based identification for volatile constituents; isolated curcumin confirmed by IR and NMR	[53]
	β-pinene	flower, leaf	Not reported	Not reported	[9]
		rhizome, root tuber	GC-MS fingerprinting with chemometric analysis	GC-MS identification based on mass-spectral/retention information	[57]
		rhizome	Gas chromatography (GC)	GC-based identification; detailed structural confirmation not reported	[59]
		rhizome	HS-SPME-GC-MS	GC-MS identification using spectral/retention information	[62]
	borneol	rhizome	Gas chromatography (GC)	GC-based identification; detailed structural confirmation not reported	[59]

Phytochemical group	Compound	Plant part	Identification method / analytical basis	Level of structural confirmation	Reference(s)
	camphene	root tuber	GC-MS fingerprinting with chemometric analysis	GC-MS identification based on mass-spectral/retention information	[57]
	camphor	rhizome	HS-SPME-GC-MS	GC-MS identification using spectral/retention information	[62]
	cis-p-menth-2,8-dienol	rhizome	GC-MS for volatile constituents; column chromatography with IR and 1H/13C NMR for isolated curcumin	GC-MS-based identification for volatile constituents; isolated curcumin confirmed by IR and NMR	[53]
	cis-p-mentha-1(7),8-dien-2-ol	rhizome	HPLC analysis of curcuminoids; GC-MS analysis of volatile compounds	Chromatographic/MS-based identification; detailed structural confirmation not reported	[61]
	cis-sabinol	rhizome	GC-MS for volatile constituents; column chromatography with IR and 1H/13C NMR for isolated curcumin	GC-MS-based identification for volatile constituents; isolated curcumin confirmed by IR and NMR	[53]
	D-limonene	rhizome, root tuber	GC-MS fingerprinting with chemometric analysis	GC-MS identification based on mass-spectral/retention information	[56]
	isopulegol	leaf	Bioassay-guided isolation; preparative HPLC; 1H/13C NMR	Isolated; structure confirmed by NMR	[50]
	limonene	leaf	GC analysis of leaf essential oils	GC-based identification; detailed structural confirmation not reported	[54]
	linalool	rhizome	Gas chromatography (GC)	GC-based identification; detailed structural confirmation not reported	[59]
	m-cymene	rhizome, root tuber	GC-MS fingerprinting with chemometric analysis	GC-MS identification based on mass-spectral/retention information	[57]
	menth-1-en-9-ol	leaf	Bioassay-guided isolation; preparative HPLC; 1H/13C NMR	Isolated; structure confirmed by NMR	[50]
	myrcene	rhizome, leaf, flower	Not reported	Not reported	[9]
			GC analysis of leaf essential oils	GC-based identification; detailed structural confirmation not reported	[54]
	o-cymene	rhizome	GC-MS for volatile constituents; column chromatography with IR and 1H/13C NMR for isolated curcumin	GC-MS-based identification for volatile constituents; isolated curcumin confirmed by IR and NMR	[53]
	p-cymen-8-ol	rhizome, root tuber	GC-MS fingerprinting with chemometric analysis	GC-MS identification based on mass-spectral/retention information	[57]

Phytochemical group	Compound	Plant part	Identification method / analytical basis	Level of structural confirmation	Reference(s)
	p-cymene	leaf	GC analysis of leaf essential oils	GC-based identification; detailed structural confirmation not reported	[54]
			GC/GC-MS analysis of leaf and rhizome essential oils	GC/GC-MS-based identification; detailed structural confirmation not reported	[60]
	sabinene	flower, leaf	Not reported	Not reported	[9]
	terpinolene	leaf	GC analysis of leaf essential oils	GC-based identification; detailed structural confirmation not reported	[54]
		leaf, rhizome	GC/GC-MS analysis of leaf and rhizome essential oils	GC/GC-MS-based identification; detailed structural confirmation not reported	[60]
		rhizome	GC-MS	GC-MS-based identification	[63]
	trans-chrysanthenyl acetate	rhizome	GC-MS for volatile constituents; column chromatography with IR and 1H/13C NMR for isolated curcumin	GC-MS-based identification for volatile constituents; isolated curcumin confirmed by IR and NMR	[53]
	tricyclene	flower	Not reported	Not reported	[9]
Sesquiterpenoids	(−)-β-sesquiphellandrene	rhizome, root tuber	GC-MS fingerprinting with chemometric analysis	GC-MS identification based on mass-spectral/retention information	[57]
	(E)-α-santalal	rhizome	HS-SPME-GC-MS	GC-MS identification using spectral/retention information	[62]
	(E)-atlantone	rhizome	HS-SPME-GC-MS	GC-MS identification using spectral/retention information	[62]
	(E)-β-farnesene	rhizome, root tuber	GC-MS fingerprinting with chemometric analysis	GC-MS identification based on mass-spectral/retention information	[57]
		rhizome	HPLC analysis of curcuminoids; GC-MS analysis of volatile compounds	Chromatographic/MS-based identification; detailed structural confirmation not reported	[61]
		rhizome	HS-SPME-GC-MS	GC-MS identification using spectral/retention information	[62]
	(E)-γ-atlantone	rhizome	HS-SPME-GC-MS	GC-MS identification using spectral/retention information	[62]
	7-epi-sesquithujene	rhizome, root tuber	GC-MS fingerprinting with chemometric analysis	GC-MS identification based on mass-spectral/retention information	[57]
		rhizome	HS-SPME-GC-MS	GC-MS identification using spectral/retention information	[62]
	8,12-epoxygermacra-1(10),4,7,11-tetraene	leaf	Bioassay-guided isolation; preparative HPLC; 1H/13C NMR	Isolated; structure confirmed by NMR	[50]

Phytochemical group	Compound	Plant part	Identification method / analytical basis	Level of structural confirmation	Reference(s)
	α-acorenol	rhizome	HS-SPME-GC-MS	GC-MS identification using spectral/retention information	[62]
	α-atlantone	rhizome	GC-MS	GC-MS-based identification	[64]
	α-cedrene	rhizome	GC-MS for volatile constituents; column chromatography with IR and 1H/13C NMR for isolated curcumin	GC-MS-based identification for volatile constituents; isolated curcumin confirmed by IR and NMR	[53]
	α-humulene	rhizome, root tuber	GC-MS fingerprinting with chemometric analysis	GC-MS identification based on mass-spectral/retention information	[57]
		rhizome	HS-SPME-GC-MS	GC-MS identification using spectral/retention information	[62]
	α-santalene	rhizome	Not reported	Not reported	[32]
	α-turmerone	rhizome	Not reported	Not reported	[32]
		leaf, rhizome	GC/GC-MS analysis of leaf and rhizome essential oils	GC/GC-MS-based identification; detailed structural confirmation not reported	[60]
		rhizome, tuber	Pressurized liquid extraction followed by GC-MS	GC-MS identification/quantification	[65]
	α-zingiberene	rhizome	GC-MS and GC-FID	GC-MS/retention-data based identification	[55]
		root tuber	Isolation from root tuber; detailed analytical method not reported in accessible abstract	Isolated; detailed structural confirmation not reported	[56]
		rhizome	HS-SPME-GC-MS	GC-MS identification using spectral/retention information	[62]
	ar-curcumene	rhizome	Not reported	Not reported	[32]
		rhizome	GC-MS for volatile constituents; column chromatography with IR and 1H/13C NMR for isolated curcumin	GC-MS-based identification for volatile constituents; isolated curcumin confirmed by IR and NMR	[53]
		rhizome	HS-SPME-GC-MS	GC-MS identification using spectral/retention information	[62]
		rhizome, root tuber	Pressurized liquid extraction followed by GC-MS	GC-MS identification/quantification	[65]
	ar-turmerol	rhizome	HS-SPME-GC-MS	GC-MS identification using spectral/retention information	[62]
			Accelerated/fractionated solvent extraction; detailed identification method not reported	Not reported	[65]
	ar-turmerone	rhizome	Not reported	Not reported	[32]
		rhizome	HPLC-ESI-MS/MS and GNPS molecular networking	Putative annotation based on MS/MS fragmentation and molecular networking	[45]
		rhizome	Secondary review	Secondary review	[46]

Phytochemical group	Compound	Plant part	Identification method / analytical basis	Level of structural confirmation	Reference(s)
		rhizome	GC-MS for volatile constituents; column chromatography with IR and 1H/13C NMR for isolated curcumin	GC-MS-based identification for volatile constituents; isolated curcumin confirmed by IR and NMR	[53]
		rhizome	GC-MS and GC-FID	GC-MS/retention-data based identification	[55]
		rhizome, root tuber	GC-MS fingerprinting with chemometric analysis	GC-MS identification based on mass-spectral/retention information	[57]
		leaf, rhizome	GC/GC-MS analysis of leaf and rhizome essential oils	GC/GC-MS-based identification; detailed structural confirmation not reported	[60]
		rhizome	HPLC analysis of curcuminoids; GC-MS analysis of volatile compounds	Chromatographic/MS-based identification; detailed structural confirmation not reported	[61]
		rhizome	HS-SPME-GC-MS	GC-MS identification using spectral/retention information	[62]
		rhizome	GC-MS	GC-MS-based identification	[64]
		rhizome	GC and GC-MS analysis of essential oils	GC-MS/retention-based identification	[67]
		rhizome	GC-MS analysis of essential oils	GC-MS-based identification	[68]
	β-bisabolene	rhizome	GC-MS for volatile constituents; column chromatography with IR and 1H/13C NMR for isolated curcumin	GC-MS-based identification for volatile constituents; isolated curcumin confirmed by IR and NMR	[53]
		rhizome, root tuber	GC-MS fingerprinting with chemometric analysis	GC-MS identification based on mass-spectral/retention information	[57]
		rhizome	HPLC analysis of curcuminoids; GC-MS analysis of volatile compounds	Chromatographic/MS-based identification; detailed structural confirmation not reported	[61]
		rhizome	HS-SPME-GC-MS	GC-MS identification using spectral/retention information	[62]
	β-caryophyllene	rhizome	GC-MS for volatile constituents; column chromatography with IR and 1H/13C NMR for isolated curcumin	GC-MS-based identification for volatile constituents; isolated curcumin confirmed by IR and NMR	[53]
			GC-MS and GC-FID	GC-MS/retention-data based identification	[55]
		root tuber	Not reported	Isolated; detailed structural confirmation not reported	[56]

Phytochemical group	Compound	Plant part	Identification method / analytical basis	Level of structural confirmation	Reference(s)
	β-cedrene	rhizome	GC-MS for volatile constituents; column chromatography with IR and 1H/13C NMR for isolated curcumin	GC-MS-based identification for volatile constituents; isolated curcumin confirmed by IR and NMR	[53]
			HS-SPME-GC-MS	GC-MS identification using spectral/retention information	[62]
	β-curcumene	rhizome	GC-MS analysis of essential oils	GC-MS-based identification	[69]
	β-elemene	rhizome	GC-MS for volatile constituents; column chromatography with IR and 1H/13C NMR for isolated curcumin	GC-MS-based identification for volatile constituents; isolated curcumin confirmed by IR and NMR	[53]
			HS-SPME-GC-MS	GC-MS identification using spectral/retention information	[62]
	β-selinene	rhizome	HS-SPME-GC-MS	GC-MS identification using spectral/retention information	[62]
	β-sesquiphellandrene	rhizome	GC-MS for volatile constituents; column chromatography with IR and 1H/13C NMR for isolated curcumin	GC-MS-based identification for volatile constituents; isolated curcumin confirmed by IR and NMR	[53]
		rhizome	GC-MS and GC-FID	GC-MS/retention-data based identification	[55]
		rhizome, root tuber	GC-MS fingerprinting with chemometric analysis	GC-MS identification based on mass-spectral/retention information	[57]
		rhizome	HS-SPME-GC-MS	GC-MS identification using spectral/retention information	[62]
		rhizome	GC-MS	GC-MS-based identification	[63]
	β-turmerone	rhizome	Not reported	Not reported	[32]
		rhizome	GC-MS for volatile constituents; column chromatography with IR and 1H/13C NMR for isolated curcumin	GC-MS-based identification for volatile constituents; isolated curcumin confirmed by IR and NMR	[53]
		rhizome	GC-MS and GC-FID	GC-MS/retention-data based identification	[55]
		rhizome, root tuber	GC-MS fingerprinting with chemometric analysis	GC-MS identification based on mass-spectral/retention information	[57]
		leaf, rhizome	GC/GC-MS analysis of leaf and rhizome essential oils	GC/GC-MS-based identification; detailed structural confirmation not reported	[60]
		rhizome	HS-SPME-GC-MS	GC-MS identification using spectral/retention information	[62]

Phytochemical group	Compound	Plant part	Identification method / analytical basis	Level of structural confirmation	Reference(s)
	β-vatirenene	rhizome	GC-MS for volatile constituents; column chromatography with IR and 1H/13C NMR for isolated curcumin	GC-MS-based identification for volatile constituents; isolated curcumin confirmed by IR and NMR	[53]
	β-ylangene	rhizome	HS-SPME-GC-MS	GC-MS identification using spectral/retention information	[62]
	bisabolone	rhizome	HS-SPME-GC-MS	GC-MS identification using spectral/retention information	[62]
	bisacurone	root tuber	Isolation from root tuber	Isolated; detailed structural confirmation not reported	[56]
		rhizome; root tuber	Isolation; 1D/2D NMR spectroscopy; ECD for absolute configuration where applicable	Isolated; structure elucidated by NMR; absolute configuration by ECD where applicable	[70]
	bisacurone A	root tuber	Isolation from root tuber	Isolated; detailed structural confirmation not reported	[56]
		rhizome; root tuber	Isolation; 1D/2D NMR spectroscopy; ECD for absolute configuration where applicable	Isolated; structure elucidated by NMR; absolute configuration by ECD where applicable	[70]
	bisacurone B	root tuber	Isolation from root tuber	Isolated; detailed structural confirmation not reported	[56]
		rhizome; root tuber	Isolation; 1D/2D NMR spectroscopy; ECD for absolute configuration where applicable	Isolated; structure elucidated by NMR; absolute configuration by ECD where applicable	[70]
	caryophyllene oxide	rhizome, root tuber	GC-MS fingerprinting with chemometric analysis	GC-MS identification based on mass-spectral/retention information	[57]
		rhizome	HS-SPME-GC-MS	GC-MS identification using spectral/retention information	[61]
	cis-α-bergamotene	rhizome, root tuber	GC-MS fingerprinting with chemometric analysis	GC-MS identification based on mass-spectral/retention information	[57]
		rhizome	HS-SPME-GC-MS	GC-MS identification using spectral/retention information	[61]
	cis-sesquisabinene hydrate	rhizome	HS-SPME-GC-MS	GC-MS identification using spectral/retention information	[62]
	cryptomeridiol	rhizome	HS-SPME-GC-MS	GC-MS identification using spectral/retention information	[62]
	curcumenol	rhizome	GC-MS for volatile constituents; column chromatography with IR and 1H/13C NMR for isolated curcumin	GC-MS-based identification for volatile constituents; isolated curcumin confirmed by IR and NMR	[53]
			HS-SPME-GC-MS	GC-MS identification using spectral/retention information	[62]

Phytochemical group	Compound	Plant part	Identification method / analytical basis	Level of structural confirmation	Reference(s)
	curcuphenol	rhizome, root tuber	GC-MS fingerprinting with chemometric analysis	GC-MS identification based on mass-spectral/retention information	[57]
	curdione	rhizome	HS-SPME-GC-MS	GC-MS identification using spectral/retention information	[62]
	δ-cadinene	rhizome	HS-SPME-GC-MS	GC-MS identification using spectral/retention information	[62]
	epicurzerenone	rhizome	HS-SPME-GC-MS	GC-MS identification using spectral/retention information	[62]
	farnesol	leaf	GC analysis of leaf essential oils	GC-based identification; detailed structural confirmation not reported	[54]
	furanodienone (8,12-epoxygermacra-1(10), 4,7,11-tetraen-6-one	leaf	Bioassay-guided isolation; preparative HPLC; 1H/13C NMR	Isolated; structure confirmed by NMR	[50]
	γ-curcumene	rhizome, root tuber	GC-MS fingerprinting with chemometric analysis	GC-MS identification based on mass-spectral/retention information	[57]
	germacrene B	rhizome	HS-SPME-GC-MS	GC-MS identification using spectral/retention information	[62]
	germacron-13-al	rhizome	Isolation; spectroscopic analysis (NMR/MS)	Isolated; structure elucidated by spectroscopy	[71]
	germacrone	rhizome	HS-SPME-GC-MS	GC-MS identification using spectral/retention information	[62]
	nerolidol	rhizome, root tuber	GC-MS fingerprinting with chemometric analysis	GC-MS identification based on mass-spectral/retention information	[57]
	sesquisabinene	rhizome	HPLC analysis of curcuminoids; GC-MS analysis of volatile compounds	Chromatographic/MS-based identification; detailed structural confirmation not reported	[61]
	trans-α-bergamotene	rhizome	HPLC analysis of curcuminoids; GC-MS analysis of volatile compounds	Chromatographic/MS-based identification; detailed structural confirmation not reported	[61]
	trans-nuciferol	rhizome	HS-SPME-GC-MS	GC-MS identification using spectral/retention information	[62]
	turmerol	rhizome	GC-MS	GC-MS-based identification	[64]
	turmerone Q	rhizome	Isolation; 1D/2D NMR spectroscopy; ECD for absolute configuration where applicable	Isolated; structure elucidated by NMR; absolute configuration by ECD where applicable	[70]
	turmeronol A	root tuber	Isolation and chromatographic purification; spectroscopic analysis (NMR/MS)	Isolated; structure elucidated by spectroscopy	[40]
		rhizome	HPLC-ESI-MS/MS and GNPS molecular networking	Putative annotation based on MS/MS fragmentation and molecular networking	[45]

Phytochemical group	Compound	Plant part	Identification method / analytical basis	Level of structural confirmation	Reference(s)
		root tuber	Isolation from root tuber	Isolated; detailed structural confirmation not reported	[56]
		rhizome	Isolation; spectroscopic analysis (NMR/MS)	Isolated; structure elucidated by spectroscopy	[72]
	turmeronol B	rhizome	Isolation; spectroscopic analysis (NMR/MS)	Isolated; structure elucidated by spectroscopy	[72]
	zingiberenol	rhizome, root tuber	GC-MS fingerprinting with chemometric analysis	GC-MS identification based on mass-spectral/retention information	[57]
		rhizome	HS-SPME-GC-MS	GC-MS identification using spectral/retention information	[62]
Diterpenoids	(E)-labda-8(17),12-dien-15,16-dial	leaf	Bioassay-guided fractionation/isolation; spectroscopic identification	Isolated constituents; structural identification reported	[73]
	(E,E,E)-3,7,11,15- tetramethylhexadeca-1,3,6,10,14-pentaene	rhizome	GC-MS analysis of rhizome essential oil	GC-MS-based identification	[74]
	(E,E)- 3,7,11,15-tetramethylhexadeca-1,6,10,14-tetraen-3-ol	rhizome	GC-MS analysis of rhizome essential oil	GC-MS-based identification	[74]
	2,6,11,15-tetramethyl-hexadeca-2,6,8,10,14-pentaene	rhizome	GC-MS analysis of rhizome essential oil	GC-MS-based identification	[74]
	coronadiene	leaf	Bioassay-guided isolation; preparative HPLC; 1H/13C NMR	Isolated; structure confirmed by NMR	[50]
	geranyl-p-cumene	rhizome	GC-MS for volatile constituents; column chromatography with IR and 1H/13C NMR for isolated curcumin	GC-MS-based identification for volatile constituents; isolated curcumin confirmed by IR and NMR	[53]
	phytol	rhizome	GC-MS and LC-ESI-MS metabolic profiling	MS-based metabolite identification/quantification	[75]
Fatty acids and lipid derivatives	8,11-octadecadienoic acid, methyl ester	rhizome	GC-MS and LC-ESI-MS metabolic profiling	MS-based metabolite identification/quantification	[75]
	α-linolenic acid	rhizome, root tuber	GC-MS-based fatty-acid profiling	GC-MS identification using mass-spectral/retention comparison	[76]
	arachidic acid	rhizome, root tuber	GC-MS-based fatty-acid profiling	GC-MS identification using mass-spectral/retention comparison	[76]
	docosanoic acid	rhizome, root tuber	GC-MS-based fatty-acid profiling	GC-MS identification using mass-spectral/retention comparison	[76]
	heptadecanoic acid	rhizome, root tuber	GC-MS-based fatty-acid profiling	GC-MS identification using mass-spectral/retention comparison	[76]
	lignoceric acid	rhizome, root tuber	GC-MS-based fatty-acid profiling	GC-MS identification using mass-spectral/retention comparison	[76]
	linoleic acid	rhizome	GC-MS and LC-ESI-MS metabolic profiling	MS-based metabolite identification/quantification	[75]
		rhizome, root tuber	GC-MS-based fatty-acid profiling	GC-MS identification using mass-spectral/retention comparison	[76]

Phytochemical group	Compound	Plant part	Identification method / analytical basis	Level of structural confirmation	Reference(s)
	oleic acid	rhizome	HPLC analysis of curcuminoids; GC-MS analysis of volatile compounds	Chromatographic/MS-based identification; detailed structural confirmation not reported	[61]
		rhizome	GC-MS and LC-ESI-MS metabolic profiling	MS-based metabolite identification/quantification	[75]
		rhizome, root tuber	GC-MS-based fatty-acid profiling	GC-MS identification using mass-spectral/retention comparison	[76]
	palmitic acid	rhizome, root tuber	HPLC analysis of curcuminoids; GC-MS analysis of volatile compounds	Chromatographic/MS-based identification; detailed structural confirmation not reported	[61]
			GC-MS and LC-ESI-MS metabolic profiling	MS-based metabolite identification/quantification	[75]
	palmitoleic acid	root tuber	GC-MS-based fatty-acid profiling	GC-MS identification using mass-spectral/retention comparison	[76]
	pentadecanoic acid	rhizome, root tuber	GC-MS-based fatty-acid profiling	GC-MS identification using mass-spectral/retention comparison	[76]
	stearic acid	rhizome	HPLC analysis of curcuminoids; GC-MS analysis of volatile compounds	Chromatographic/MS-based identification; detailed structural confirmation not reported	[61]
		rhizome	GC-MS and LC-ESI-MS metabolic profiling	MS-based metabolite identification/quantification	[75]
		rhizome, root tuber	GC-MS-based fatty-acid profiling	GC-MS identification using mass-spectral/retention comparison	[76]
Sterols and steroidal compound	20-oxopregn-16-en-12-yl acetate	rhizome	Isolation and chromatographic purification; spectroscopic analysis	Isolated; structure elucidated by spectroscopy	[52]
	β-sitosterol	rhizome	Isolation and chromatographic purification; spectroscopic analysis	Isolated; structure elucidated by spectroscopy	[52]
	gitoxigenin	rhizome	Isolation and chromatographic purification; spectroscopic analysis	Isolated; structure elucidated by spectroscopy	[52]
	stigmasterol	rhizome	Isolation and chromatographic purification; spectroscopic analysis	Isolated; structure elucidated by spectroscopy	[52]
Triterenoids	hop-17(21)-en-3-ol	rhizome	Secondary review	Secondary review	[77]
	hop-17(21)-en-3-yl acetate	rhizome	Secondary review	Secondary review	[77]
	hopenone I	rhizome	Secondary review	Secondary review	[77]
Alkaloids and other nitrogen-containing compounds	2,3,5,6-tetramethylpyrazine	root tuber	GC-MS fingerprinting with chemometric analysis	GC-MS identification based on mass-spectral/retention information	[57]
	2-(2'-methyl-1'-propenyl)-4,6-dimethyl-7-hydroxyquinoline	root tuber	Isolation from root tuber	Isolated; detailed structural confirmation not reported	[56]
Other compounds	2-tridecanol	rhizome	HS-SPME-GC-MS	GC-MS identification using spectral/retention information	[62]
	2-undecanone	rhizome	HS-SPME-GC-MS	GC-MS identification using spectral/retention information	[62]

Phytochemical group	Compound	Plant part	Identification method / analytical basis	Level of structural confirmation	Reference(s)
	3,3,5,5-tetramethylcyclopentene	rhizome, root tuber	GC-MS fingerprinting with chemometric analysis	GC-MS identification based on mass-spectral/retention information	[57]
		rhizome	HS-SPME-GC-MS	GC-MS identification using spectral/retention information	[62]
	hemellitol	rhizome	GC-MS for volatile constituents; column chromatography with IR and 1H/13C NMR for isolated curcumin	GC-MS-based identification for volatile constituents; isolated curcumin confirmed by IR and NMR	[53]
	phthalic acid	rhizome	HPLC analysis of curcuminoids; GC-MS analysis of volatile compounds	Chromatographic/MS-based identification; detailed structural confirmation not reported	[61]
	undecanol	leaf	GC/GC-MS analysis of leaf and rhizome essential oils	GC/GC-MS-based identification; detailed structural confirmation not reported	[60]

Abbreviations: GC, gas chromatography; GC-FID, gas chromatography-flame ionization detection; GC-MS, gas chromatography-mass spectrometry; HPLC, high-performance liquid chromatography; HR, high-resolution; ESI, electrospray ionization; QTOF, quadrupole time-of-flight; CID, collision-induced dissociation; GNPS, Global Natural Products Social Molecular Networking; NMR, nuclear magnetic resonance; RI, retention index; FAME, fatty acid methyl ester.

Notes: Each citation is described separately when a compound is supported by more than one reference. “Secondary review” indicates that the cited source is a review article rather than a primary analytical study. “Not reported” indicates that the relevant analytical or structural-confirmation information was not reported or could not be verified from the cited source.
